# A genomic glimpse of aminoacyl-tRNA synthetases in malaria parasite *Plasmodium falciparum*

**DOI:** 10.1186/1471-2164-10-644

**Published:** 2009-12-31

**Authors:** Tarun Kumar Bhatt, Charu Kapil, Sameena Khan, Mohamad Aman Jairajpuri, Vinay Sharma, Daniele Santoni, Francesco Silvestrini, Elisabetta Pizzi, Amit Sharma

**Affiliations:** 1 Structural and Computational Biology Group, International Centre for Genetic Engineering and Biotechnology, New Delhi 110 067, India; 2 Department of Biosciences, Jamia Millia Islamia University, Jamia Nagar, New-Delhi 110 025, India; 3 Department of Bioscience and Biotechnology, Banasthali Vidyapith University, Banasthali, Rajasthan 304 022, India; 4 Barcelona Institute for Research in Biomedicine, Barcelona Science Park, C/Samitier 1-5, Barcelona 08015, Catalonia, Spain; 5 Dipartimento di Malattie Infettive, Parassitarie ed Immunomediate, Istituto Superiore di Sanità, Viale Regina Elena, 299, 00161 Rome, Italy

## Abstract

**Background:**

*Plasmodium *parasites are causative agents of malaria which affects >500 million people and claims ~2 million lives annually. The completion of *Plasmodium *genome sequencing and availability of PlasmoDB database has provided a platform for systematic study of parasite genome. Aminoacyl-tRNA synthetases (*aaRS*s) are pivotal enzymes for protein translation and other vital cellular processes. We report an extensive analysis of the *Plasmodium falciparum *genome to identify and classify *aaRSs *in this organism.

**Results:**

Using various computational and bioinformatics tools, we have identified 37 *aaRS*s in *P. falciparum*. Our key observations are: (i) fraction of proteome dedicated to *aaRS*s in *P. falciparum *is very high compared to many other organisms; (ii) 23 out of 37 *Pf-aaRS *sequences contain signal peptides possibly directing them to different cellular organelles; (iii) expression profiles of *Pf-aaRSs *vary considerably at various life cycle stages of the parasite; (iv) several *PfaaRSs *posses very unusual domain architectures; (v) phylogenetic analyses reveal evolutionary relatedness of several parasite *aaRS*s to bacterial and plants *aaRSs*; (vi) three dimensional structural modelling has provided insights which could be exploited in inhibitor discovery against parasite *aaRSs*.

**Conclusion:**

We have identified 37 *Pf-aaRSs *based on our bioinformatics analysis. Our data reveal several unique attributes in this protein family. We have annotated all 37 *Pf-aaRSs *based on predicted localization, phylogenetics, domain architectures and their overall protein expression profiles. The sets of distinct features elaborated in this work will provide a platform for experimental dissection of this family of enzymes, possibly for the discovery of novel drugs against malaria.

## Background

Aminoacylation is the process of adding an aminoacyl group to the 3' end (CCA) of the tRNA molecule. tRNA is aminoacylated with a specific amino acid by aminoacyl-tRNA synthetase (*aaRS*s). *aaRS*s are responsible for attaching correct amino acid onto the cognate tRNA molecule in a two-step reaction. The amino acid is first activated with ATP forming an aminoacyladenylate intermediate. Once activated, this amino acid is transferred to the 3' end of its corresponding tRNA molecule to be processed during protein synthesis. All *aaRSs *require divalent cation MgCl_2 _for their aminoacylation reaction [1,2].

Reaction:

1. amino acid + ATP → aminoacyl-AMP + PPi

2. aminoacyl-AMP + tRNA → aminoacyl-tRNA + AMP

The *aaRS*s are divided into two major classes based on structural topology of their active sites. Class I *aaRS*s represent 11 amino acids, including Arg, Cys, Gln, Glu, Ile, Leu, Lys, Met, Val, Trp and Tyr. Class II *aaRS*s includes 10 amino acids - Ala, Asp, Asn, Gly, His, Lys, Phe, Pro, Ser and Thr. Core domains of class I enzymes are characterized by a Rossmann fold which consists of α-helices and β-pleated sheets. This domain contains two conserved motifs ('HIGH' and 'KMSKS') which are directly involved in ATP binding. Catalytic domain of class II enzymes has a unique fold with a central core of anti-parallel β strands flanked by α helices [3]. There are three weakly conserved motifs, two of them are involved in ATP binding while the third one plays a role in homo dimerization. Class I enzymes bind ATP in an extended conformation while class II do so in a bent conformation. The two *aaRS *classes have different modes of aminoacylation - class I enzymes aminoacylate the 2'OH of the cognate tRNA whereas class II enzymes aminoacylate 3'OH of the tRNA (with the exception of *PheRS*) [4]. All known *aaRS*s are multidomain proteins with complex modular architectures [5]. In addition, eukaryotic *aaRSs *are distinguished by the presence of appended domains at either the N- or C-terminus which are generally absent from their bacterial/archaeal counterparts [6]. These appendages to the catalytic cores of several *aaRSs *are non-catalytic and instead function to mediate protein- protein interactions or act as general RNA-binding domains [7-9].

In mammalian cells, some *aaRS*s are present as a larger multi- *aaRS *complex (MSC) composed of nine synthetases (arginyl-, aspartyl-, glutamyl-, glutaminyl-, leucyl-, lysyl-, isoleucyl-, methionyl- and prolyl-tRNA synthetases) [10-12]. The MSC is composed of a mixture of class I and class II *aaRS*s along with three non- *aaRS *proteins p38, p43 and p18. It is not clear why certain *aaRS*s exist as a complex while some are in free form. MSC might help in efficient protein synthesis by preventing mixing of charged tRNAs with cellular pool and by increasing local concentration of tRNA near the site of protein synthesis [13].

The accuracy of tRNA aminoacylation reaction is critical in ensuring fidelity in protein translation [14]. To achieve this accuracy, some *aaRS *enzymes possess a proofreading (editing) mechanism that hydrolyzes tRNAs aminoacylated with the non-cognate amino acid [15]. For example, editing domains may be found attached to alanyl-tRNA synthetase (*AlaRS*), leucyltRNA synthetase (*LeuRS*) and so on [16-21]. In other cases, the editing domain is not attached to *aaRS *but rather functions as an individual protein [22,23]. For example, YbaK protein from *Haemophilus influenza *is capable of efficiently editing Cys-tRNA^Pro ^[24]. *ThrRS *has been shown to have another editing domain called NTD which can cleave the bond between D-amino acid and tRNA [25].

Recently it has been shown that *aaRS*s are not only involved in protein synthesis but also perform many non-catalytic and non-canonical roles in RNA processing/trafficking, apoptosis, rRNA synthesis, angiogenesis and inflammation [26-30]. These versatile properties of *aaRS*s are the outcome of their differential cellular localization, nucleic acid binding properties, protein-protein interactions and collaboration (fusion) with additional domains. In case of malaria parasite, apicoplast proteins and pathways have already received particular attention as drug targets [31]. In this work we present a study of *aaRS*s from *P. falciparum *- the most virulent agent of human malaria. Our aim for this study was to use bioinformatics tools to (a) discover special and unusual modules present in parasite *aaRSs *which are potentially absent from human homologues, and (b) to identify potential new drug targets based on this protein family.

## Results and Discussion

### Sequence extraction and analysis

We exploited current annotation available in PlasmoDB [32] to identify the repertoire of *aaRS*s in *P. falciparum *genome. According to Enzyme Commission (EC) 37 proteins in PlasmoDB (see additional file 1) are annotated as belonging to the EC group 6.1.1. (EC number provided for *aaRS*s). Although in many cases current annotations allow an assignment to Class I or II of *aaRS*s, for some annotations are still preliminary. Due to this, we used Hidden Markov Models (HMMs) for identifying *aaRSs *in *P. falciparum*. For each *aaRS *a set of known sequences was utilized to construct 20 HMMs (see methods for details). For each database search a score distribution was obtained and 4 cutoffs were considered to identify *aaRS*. Results are reported in Table 1. We observed that 2 proteins annotated as belonging to EC group 6.1.1.- in PlasmoDB are not found by HMMs - PF14_0401 annotated as *MetRS *is instead a generic tRNA binding protein as elucidated in the genome re-annotation process, while the second one (PFC0470w) is still mis-annotated as *ValRS*. A total of 18 *Pf-aaRS*s can be classified within the 10 *aaRS*s that define class I. All members of this class are represented in the *P. falciparum *proteome. The annotations of these sequences are summarized in additional file 1. Similar to class I *Pf-aaRSs*, the class II *Pf-aaRS*s have a total of 18 sequences for 10 different amino acid synthetases. Four genes are present in *P. falciparum *for *PheRS *but these likely encode for 1 heterodimeric and 2 monomeric versions of *PheRS*.

**Table 1:** Results of database searches by HMM models of *aaRS*^@^.

cutoff	HMM^Ala^	HMM^Arg^	HMM^Asn^	HMM^Asp^	HMM^Cys^	HMM^Gln^	HMM^Glu^	HMM^Gly^	HMM^His^	HMM^Ile^
c > 50	PF13_0354	PFL0900c	PFE0475w*	PFB0525w*	PF10_0149	PF13_0170	MAL13P1.281	PF14_0198	PF14_0428	PF13_0179

20< c < 50			PFB0525w*	PFA0145c PFE0475w*		PF13_0257*	PF13_0257*		PFI1645c	

10< c < 20			PFE0715w*							

5< c <10		PFI0680c		PFE0715w*						PFL1210w

**Cutoff**	**HMM^Leu^**	**HMM^Lys^**	**HMM^Met^**	**HMM^Phe^**	**HMM^Pro^**	**HMM^Ser^**	**HMM^Thr^**	**HMM^Trp^**	**HMM^Tyr^**	**HMM^Val^**

c > 50	PF08_0011	PF13_0262	PF10_0340	PFA0480w	PFL0670c	PF07_0073	PF11_0270	PF13_0205	MAL8P1.125	PF14_0589

20< c < 50	PFF1095w	PF14_0166	PF10_0053	PFF0180w	PFI1240c	PFL0770w		PFL2485c	PF11_0181	

10< c < 20				PFL1540c PF11_0051						

5< c <10										

* hits with more than one assignment to HMM model.

^@^Two proteins PF14_0401 and PFC0470w were not found by HMMs which were annotated as *tRNA *binding protein and *ValRS *in PlasmoDB respectively.

**Figure 1:**
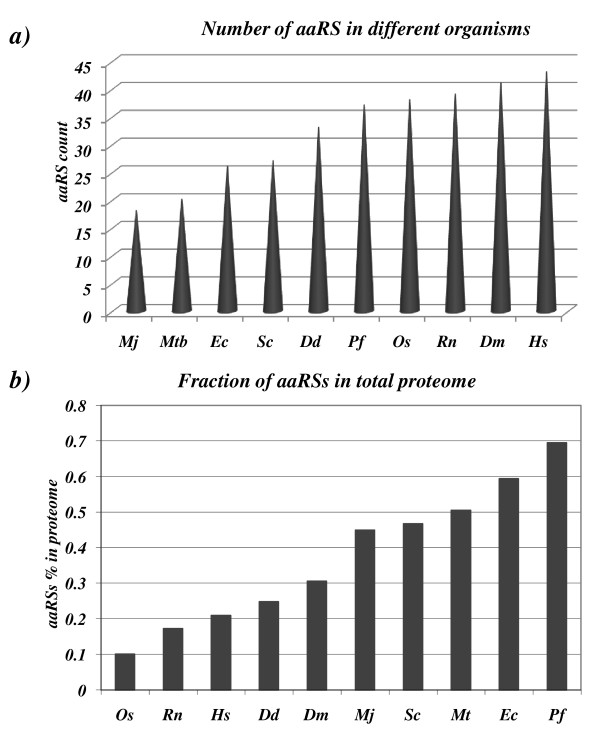
**(a) Predictied number of *aaRS*s present in *Plasmodium falciparum (Pf), Rattus norvegicus (Rn), Saccharomyces cerevisiae (Sc), Drosophila melanogaster (Dm), Homo sapiens (Hs), Oryza sativa (Os), Dictyostelium discoidium (Dd), Mycobacterium tuberculosis (Mtb), Escherichia coli (Ec)*, and *Methanocalclococcus jannaschii (Mj)***. **(b) **Diagram representing fraction of proteome (in percentage) dedicated to the *aaRS *proteins in various organisms.

**Figure 2:**
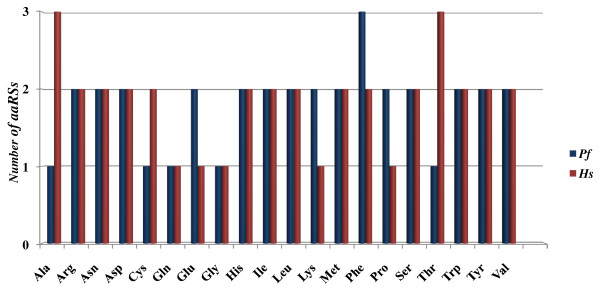
**Bar graph showing number of different *aaRSs *in *Plasmodium falciparum *and *Homo sapiens***. The number of alanyl- and threonyl- tRNA synthetases is higher in humans whereas *P. falciparum *seems richer in phe tRNA synthetases.

In order to carry out comparative analyses of *aaRS*s of *P. falciparum *with those of other species we considered *aaRS *sequences from several organisms representing three domains of life (see methods section). As expected, we found variable number of *aaRS*s in different species. *M. jannaschii *(archaebacteria) and *M. tuberculosis *(bacteria) have the lowest *aaRS*s count amongst other organisms like *E. coli*, *S. cerevisiae, D. discoidium, P. falciparum, O. sativa, R. norvegicus, D. melanogaster*, and *H. sapiens*. Human bears the highest number of *aaRS*s in this analysis (Figure 1a). Our analysis also shows that *P. falciparum *has the highest *aaRS *fraction (relative to its proteome size) when compared with bacteria, yeast and human counterparts (Figure 1b). The number of individual *aaRS *varies in different species. For example, when individual *aaRS*s from human and *P. falciparum *were compared it was evident that *AlaRS *and *ThrRS *were higher in number in humans (Figure 2). Presence of more than one copy of each *aaRS *in an organism may indicate additional biological, temporal or spatial roles for these enzymes as several *aaRS*s also perform non-canonical functions [33]. In this work we describe in detail the 37 *Pf*-*aaRS*s.

### Indirect pathways of aminoacylation

It was earlier believed that 20 *aaRS*s were necessary for the incorporation of 20 amino acids in proteins. But surprisingly, some archaea, bacteria and chloroplasts lack *GlnRS *and *AsnRS *enzymes [34-38]. Interestingly, these organisms use an alternate pathway based on tRNA dependent amino acid transformation. A non-discriminating *GluRS *charges tRNA^Gln ^with glutamic amino acid and then a second enzyme called tRNA-dependent amidotransferase (AdT) amidates glutamate to make glutamine. A corresponding reaction occurs in case of asparagine residues. In case of *P. falciparum*, occurrence of glutamate-tRNA synthetase (PF13_0257, MAL13P1.281) and amidotransferase subunit A (PFD0780w) & subunit B (PFF1395c) together indicates presence of both direct and indirect pathways for aminoacylation [39,40]. Both subunits of amidotransferase have apicoplast targeting signals suggesting an indirect pathway for aminoacylation in *P. falciparum *apicoplast. The expression of *Pf-AdT *subunit A is predicted in all life cycle stages of parasite based on proteomic and microarray data. We therefore feel that this pathway must also be active in the parasite apicoplast. We could not find sequence homologues of enzymes involved in indirect aminoacylation of cysteine residues [41-43] in the proteome of *P. falciparum*.

### The multi-synthetase complex (MSC)

In mammalian cells, some *aaRS*s are present as a larger multi-*aaRS *complex (MSC). A constituent of the MSC - protein p43 - has sequence homologue (PF14_0401 - EMAP-II-like cytokine) in *P. falciparum *although there is no evidence for presence of MSC in malaria parasites. Interestingly, p43 is not only required for stability of the MSC complex but also functions as a proinflammatory cytokine [44-46]. Role of p43 homolog in *P. falciparum *is unknown, but evidence from other organisms indicates that MSC functions in protein stability, efficient protein translation and protein elongation [47]. Sequence identity between *P. falciparum *p43 and its human homolog is ~24% and based on microarray data p43 seems to be expressed at asexual life cycle stages of *P. falciparum*. A mitochondrial targeting signal was also predicted for parasite p43 but the role of p43 in parasite remains to be explored experimentally.

### Targeting of aaRSs in the parasite

**Figure 3:**
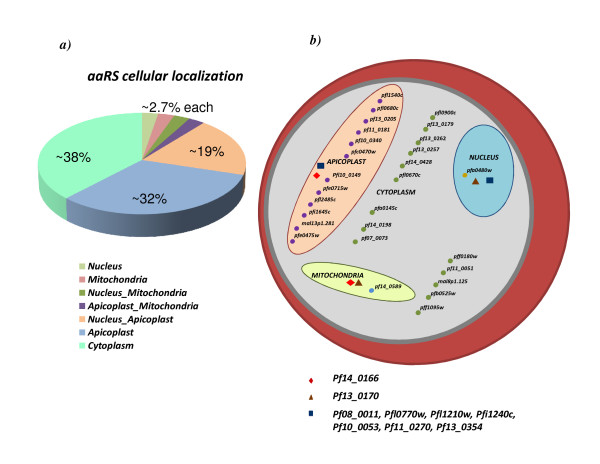
**(a) Percentage predicted distribution of *Pf*-*aaRSs *in different organelles within the parasite**. **(b) **A schematic of all *Pf-aaRS*s and their predicted cellular localization. Detailed information regarding gene IDs can be found in additional file 1. *Pf*-*aaRS*s predicted to be common between apicoplast & mitochondria, mitochondria & nucleus and apicoplast & nucleus are marked with diamond, triangle and square shapes respectively.

*aaRS*s are not only involved in protein synthesis but also in various other cellular activities including intron splicing, translational regulation and tRNA channeling. Diversified roles for *aaRS*s necessitate their presence (transit) into various cellular compartments. We therefore analyzed *P. falciparum aaRS *sequences for presence of putative signal sequences predicted by MITOPROT, PredictNLS and PATS for mitochondria, nucleus and apicoplast respectively. We found that 23 *P. falciparum aaRS*s have signal peptides, possibly for directing them to different cellular organelles. Another 14 *aaRSs *from *P. falciparum *may be resident in the parasite cytoplasm (Figure 3a). Apicoplast is known to have protein synthesis machinery which may use *aaRS*s [48]. Trafficking of nuclear encoded *aaRS*s to the apicoplast may explain why ~20 out of 37 *Pf-aaRSs *have apicoplast targeting signals. Our data indicate that out of total ~20 *Pf-aaRSs *bearing apicoplast targeting signals, ~12 *aaRS*s may be exclusive to this organelle. Others are predicted to be shared between apicoplast, nucleus and mitochondria (Figure 3b). It has been earlier shown that some tRNAs need to be aminoacylated in the nucleus before they can be exported to the cytoplasm, an observation indicating occurrence of aminoacylation reaction (mediated by *aaRSs*) inside the nucleus [49]. In *P. falciparum*, we found 10 *aaRS*s with nuclear localization signals but only one is predicted to be exclusively resident in the nucleus (PFA0480w- *PheRS*). Interestingly, we found no *Pf-aaRS *sequences with specific PEXEL (*Plasmodium *export element) motifs. This motif is found in parasite proteins that are exported beyond the parasitophorous vacuole membrane [50,51].

### Expression profiles of *P. falciparum *aaRSs

**Figure 4:**
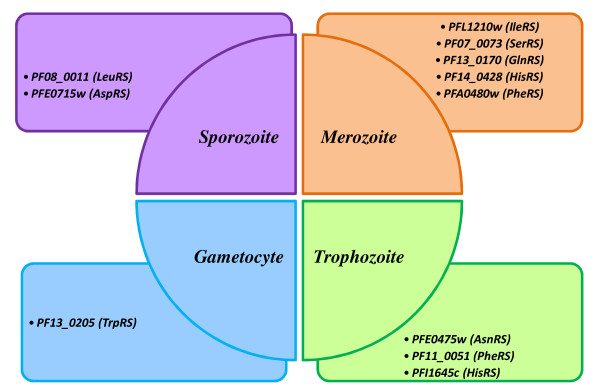
**Diagrammatic representation of *Pf-aaRS *protein expression which are specifically expressed in different life stages of the parasite based on mass spectrometry data **[82].

In order to study expression of *aaRS *during life cycle of the malaria parasite, we took advantage of available transcriptomics and proteomics data from PLASMODB. Firstly, we analyzed proteomic data from several independent experiments and compared them with transcriptomics data by Le Roch [52]. The latter sets of data were obtained using the affimetrix technology and hence provide a quantitative measure of mRNA levels in the parasite. Our results are provided in Table 2. Interestingly, we found that mRNA levels of potential apicoplast proteins (AP in the table) are lower on average (mean1 = 44.6; mean2 = 41.5; gam = 91.3; spor = 58.1) than those of potential cytoplasmic proteins (mean1 = 259; mean2 = 264.8; gam = 174.8; spor = 73.8). Proteomic data confirmed that while the cytoplasmic *aaRS *are found in almost all stages, the apicoplast *aaRS *are rarely found in the parasite. This could be in part due to experimental limits in the identification of apicoplast proteins by mass spectrometry. Indeed, when we carried out a chi-quadro test we found that proteins predicted to be targeted to apicoplast are significantly less represented (p < 10^-4^) in the sample of proteins identified by mass spectrometry. For these reasons we limited analysis of gene expression profiles only for putative cytoplasmic proteins. We considered trascriptomics data for sexual stages and asexual stages [52,53]. We considered a reduced set of the time course gene expression data (22 time points instead of 48) and normalized data by Le Roch (see methods for details). This allowed us to analyse the expression of *aaRS *genes along all the intra-erythrocytic life cycle of the parasite (Table 2). Further observations of the protein expression profiles indicated that some *aaRSs *were exclusively detected at specific stages like, *LeuRS *(PF08_0011) and *AspRS *(PFE0715w) in sporozoites; *IleRS *(PFL1210w), *SerRS *(PF07_0073), *GlnRS *(PF13_0170), *HisRS *(PF14_0428) and *PheRS *(PFA0480w) in merozoites; *AsnRS *(PFE0475w), *PheRS *(PF11_0051) and *HisRS *(PFI1645c) in trophozoites and *TrpRS *(PF13_0205) in gametocyte stages (Figure 4).

**Table 2:** Transcriptomic and proteomic data for aaRSs in P. falciparum@

ID	*aaRS*	mean1 asex	mean2 asex	Gam	spor	TG^§^	T	Me	G	Sp	Me*	Oocyst/Spor*
MAL13P1.281	AP-GluRs	4.8	10.9	39.7	6.5							
PF08_0011	AP-LeuRs	51.4	59.1	85.4	371.6					+		
PF10_0053	AP-MetRs	18.7	16.5	19	19							
PF11_0181	AP-TyrRs	13.2	2.6	0	0							
PF14_0166	AP-LysRs	60.3	48.0	166.4	48.3							
PFE0475w	AP-AsnRs	104.6	92.5	222.8	149.6		+					
PFE0715w	AP-AspRs	17.2	26.3	21.1	0					+		
PFI0680c	AP-ArgRs	15.7	17.9	34.7	1.1							
PFI1240c	AP-ProRs	10.8	14.0	19.9	4.2							
PFI1645c	AP-HisRs	27.4	21.2	8.7	15		+					
PFL0770w	AP-SerRs	117.6	110.9	431.3	69							
PFL1210w	AP-IleRs	124.0	109.5	132.3	71			+				
PFL1540c	AP-PheRs	13.7	9.7	6	0.1							

mean		44.6	41.5	91.3	58.1							

PF11_0270	ThrRs	580.0	582.1	181.2	154.6	+	+	+	+	+	+	+
PF13_0354	AlaRs	123.6	129.9	75.5	27.5	+					+	+
PF10_0149	CysRs	138.1	115.6	29.6	44.5	+						+
MAL8P1.125	TyrRs	225.6	260.0	134.3	259.2	+	+	+	+	+	+	+
PF07_0073	SerRs	190.9	193.8	364.2	22.5	+		+				+
PF10_0340	MetRs	389.3	387.5	479.9	3.4	+	+	+			+	+
PF11_0051	PheRs	126.2	126.5	46.9	1.6	+	+					
PF13_0170	GlnRs	558.8	570.8	272.3	214.7	+		+				
PF13_0179	IleRs	192.1	188.1	152.8	0	+	+	+	+	+	+	+
PF13_0205	TrpRs	112.6	189.4	42.8	171.8	+			+			
PF13_0257	GluRs	330.0	334.5	205.6	139	+	+	+	+		+	+
PF13_0262	LysRs	1048.8	971.6	591	281.9	+	+	+	+	+	+	+
PF14_0198	GlyRs	93.4	80.1	102.4	0	+	+	+			+	+
PF14_0428	HisRs	35.4	51.1	0	0	+		+			+	
PF14_0589	ValRs	48.0	33.2	5.8	0	+		+		+	+	+
PFA0145c	AspRs	359.2	359.2	361	21.4	+	+	+				
PFA0480w	PheRs	49.5	20.2	28.4	10.4	+		+			+	
PFB0525w	AsnRs	575.3	686.7	493.1	106.2	+	+	+	+		+	+
PFL0670c	ProRs	105.4	108.8	50.	5 0		+	+	+	+	+	+
PFL0900c	ArgRs	118.3	120.8	37.1	76.2	+	+	+		+	+	
PFL2485c	TrpRs	38.2	51.1	16.5	15.9							

Mean		259.0	264.8	174.8	73.8							

^@ ^Gene expression data are by Le Roch[52]. Mean1 and mean2 asex refer to mean values of mRNA abundance along asexual stages synchronized by sorbitol and by temperature respectively. In the last two columns, mass spectrometry data from purified merozoites (Me*; Leiden Malaria Group, unpublished data) and mosquito stages (Oocyst/Spor*; oocysts, oocystderived sporozoites and salivary gland sporozoites from Anopheles stephensi infected with NF54 strain of *P. falciparum*) are shown.

TG^§ ^proteomic data are obtained by Trophozoites, Gametocytes early and Gametocytes late by Lasonder lab. [88]. T (trophozoites), Me (merozoites), G (gametocytes), Sp (sporozoites) columns refer to data by multi-dimensional protein identification technology in four stages of the parasite life cycle.

### Domain architecture of *P. falciparum *aaRSs

**Figure 5:**
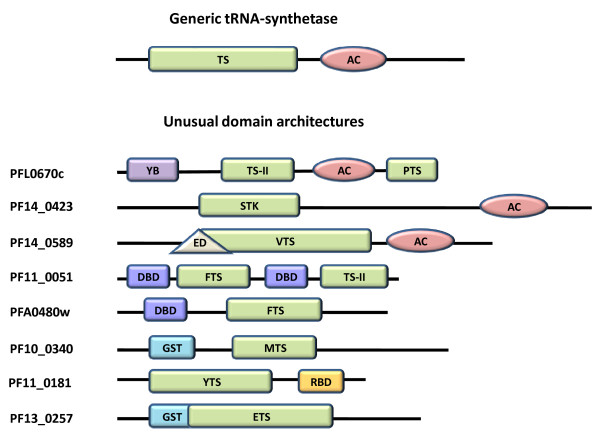
**Representation of unusual domain architectures in *Pf-aaRS*s and related proteins**. A generic *aaRS *is also shown on top. Domain name abbreviations are YB, Ybak associating domain; TS-II, class II tRNA synthetase; AC, anticodon binding site; ED, editing domain; GST, glutathione-Stransferase C-terminal region; RBD, S4 RNA binding domain; TS, tRNA synthetase core domain; STK, serine-threonine kinase; FTS, phenylalanine-tRNA synthetase; PTS, prolinetRNA synthetase; VTS, valine-tRNA synthetase; MTS, methionine-tRNA synthetase; YTS, tyrosine-tRNA synthetase; ETS, glutamate-tRNA synthetase.

*aaRS*s are multi-domain proteins typically consisting of a conserved catalytic domain and an anti-codon binding domain. In addition, some *aaRS*s have RNA binding and editing domains that cleave incorrectly aminoacylated tRNA molecules [54]. Additional functional domains may be appended to *aaRS*s in the course of biological evolution [55,56]. Careful examination of 37 identified *P. falciparum aaRS*s using Pfam database showed that most of them have a generic modular architecture that adheres to prototypical *aaRSs *(Figure 5). The remaining *P. falciparum aaRS*s or related proteins like PF14_0423 (protein having serine-threonine kinase domain in fusion with an anti-codon binding module) have complex domain architectures. In several, concatenation of unusual domains such as Ybak, GST, Ser-Thr kinase and DNA binding domains is evident (Figure 5). The functional relevance of these additional domains fused to typical *aaRS *in *P. falciparum *needs to be experimentally addressed. Intriguingly, two of the four *Pf-PheRS *subunits contain DNA binding domains (PF11_0051, PFA0480w). It is likely that the *PheRS*, in addition to its aminoacylation function, influences other cellular processes via DNA binding [57]. Consistent with its potential DNA binding property, the *P. falciparum PheRS *(PFA0480w) has a nuclear localization signal. The *CysRS *of *B. subtilis *(which also contains a DNA binding domain) is believed to play a role in initiating chromosomal replication [58]. Therefore, functional roles for *P. falciparum PheRSs *may extend from aminoacylation to DNA recognition and replication - a suggestion that requires experimental investigation. Similarly, it has been shown that GST or GST homology domains can help in complex formation of *aaRS*s with multifunctional factors (p38, p18) [56,57]. Additional data show that deletion of GST homology domain from the C-terminal region of p38 results in the dissociation of EPRS (Glutamyl-prolyl-tRNA synthetase) and *MetRS *from the MSC complex [59]. Mammalian *ValRS *associated with elongation factor subunits also contain the GST homology domain [60-62]. Thus, the presence of GST domains might be a crucial feature of *aaRS*s. *P. falciparum *proteome has two such proteins with GST domains appended to *MetRS *(PF10_0340) and *GluRS *(PF13_0257). We also found a most interesting fusion of anticodon binding domain with a serine-threonine kinase (PF14_0423) in *P. falciparum*. This unusual kinase seems to be expressed throughout the life cycle of parasite (microarray data) and interestingly is predicted to be localized to the parasite nucleus. Clearly, the presence of unusual domain fusions in *P. falciparum aaRS*s suggests multiple functional roles for many of these *P. falciparum *enzymes as has been shown in other organisms.

### Phylogenetics

**Figure 6:**
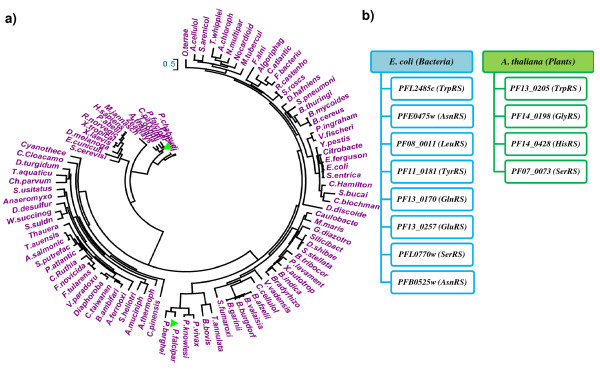
**(a) Evolutionary tree was constructed using the PHYML based on maximum likelihood method**. *P. falciparum TyrRSs *(PlasmoDB id -MAL8p1.125 and PF11_0181) are labeled as green triangles. One of the *TryRSs *(MAL8p1.125) is evolutionarily closer to *H. sapiens *whereas the other *TyrRS *(PF11_0181) is closer to *E. coli*. Total of 102 species were considered for the evolutionary analysis and were taken from three domains of life. **(b) **List of *Pf-aaRS *sequences evolutionarily closer to their *E. coli *and *A. Thaliana *counterparts.

Overall the percentage identity between matching human and *P. falciparum aaRS *domains varies from 17 to 51. Clearly, *Pf-aaRSs *which have low sequence identity with human counterparts might serve as good drug targets. In order to study evolutionary relationships of *P. falciparum aaRS*s with other species, phylogenetic trees were developed in PHYML using maximum likelihood method. For each type of *P. falciparum aaRS *a separate tree was constructed (see additional file 2). *aaRS *sequences from 102 different species were used for multiple sequence alignments. As an example, phylogenetic tree of *TyrRS *from various species (including two sequences from *P. falciparum*) was constructed. Interestingly, one *Pf-TyrRS *(MAL8P1.125) clustered with human *TyrRS *whereas the second *Pf-TyrRS *(PF11_0181) clustered with bacterial *TyrRS *indicating different evolutionary origins (Figure 6a). Based on distance matrices, several *P. f*alciparum *aaRS *sequences clustered as being closer to plants (*A. thaliana*) or to bacteria (*E. coli*) (Figure 6b). It is already known that apicomplexan parasites like *P. falciparum *house a secondary endosymbiotic plastid, possibly hijacked by lateral genetic transfer from an alga. Therefore, the *P. falciparum aaRS *sequences which are evolutionary close to bacteria and plants are likely to be the outcome of horizontal gene transfer from the plastid. *P. falciparum *contains ~12 such *aaRS *sequences which cluster with bacterial or plant sequences. Functional and structural characterization of these bacterial/plant-like *aaRS *may be relevant in focusing efforts at using *aaRS *as drug targets.

### Homology modeling and structure comparisons

**Figure 7:**
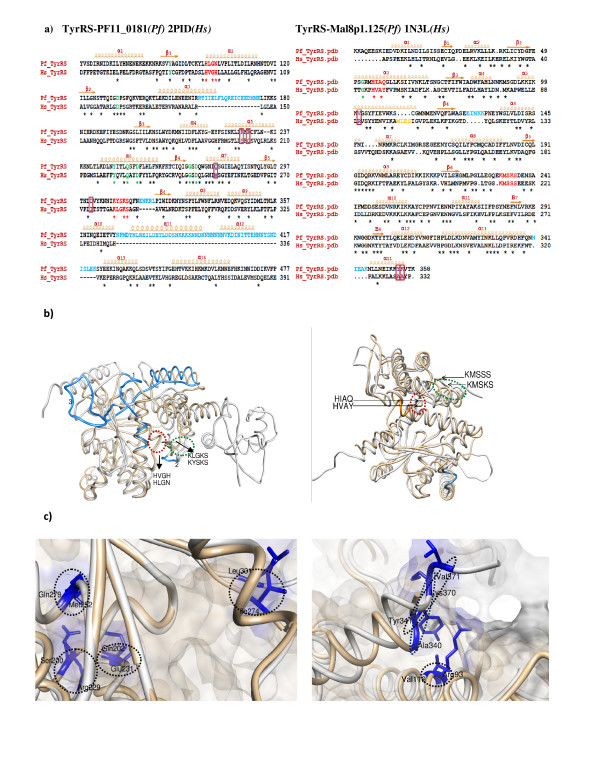
**Left and right panels of the figure represent sequence and structural comparison of bacterial type *Plasmodium TyrRS *(PF11_0181) with human mitochondrial *TyrRS *(2PID) and the cytosolic *Plasmodium TyrRS *(Mal8p1.125) with human cytosolic *TyrRS *(1N3L)**. **a) **A structure-based sequence alignment of the catalytic domain of *Plasmodium TyrRSs *with human *TyrRSs*. Insertions in *Pf *and human sequences are colored in light blue and orange respectively. Class I synthetase conserved motifs are colored red. Residues involved in tRNA recognition and catalysis are indicated in green (same residues in P*f *and H*s*) and violet & boxed (different in P*f *and H*s*). The secondary structural elements are shown above the sequence alignments. Conserved residues are indicated by asterisk below the sequence alignment. **(b) **Superposition of *Pf-TyrRS *and *Hs-TyrRS *depicting the structural differences. *Pf-Tyr *is colored grey and *Hs-TyrRS *is colored tan. Insertions in *Pf-TyrRSs *are highlighted in blue whereas *Hs*-*TyrRS *insertions are in orange. Motif 1 in *Pf *(PF11_0181 - HLGN and Mal8p1.125 - HIAQ) and *Hs *(2PID - HVGH and 1N3L - HVAY) *TyrRSs *has been encircled red whereas Motif 2 in P*f *(PF11_0181 - KLGKS and Mal8p1.125 - KMSKS) and *Hs *(2PID - KYSKS and 1N3L - KMSSS) is encircled green. **(c) **Snapshot of the active sites of *Pf *and *Hs TyrRS*s (superimposed) structures. Non-conserved active site residues colored violet are encircled.

To date, no crystal structures have been obtained for any *aaRS *from *P. falciparum*. Hence, we performed homology modeling of several *P. falciparum aaRSs *using homologous structures available in PDB. Known structural templates (≥ 40% identity) were used for molecular modeling of several *P. falciparum aaRSs *including the two *TyrRSs *(PF11_0181, MAL8P1.125), the *PheRS *(PFA0480w), *ThrRS *(PF11_0270), *LysRS *(PF13_0262), *MetRS *(PF10_0340) and *TrpRS *(PF13_0205). The program Align2D (sequence alignment module in Modeller) was used to perform dynamic programming-based global alignments of the target and template sequences. This program uses variable gap penalty for structural loops and core regions using information derived from template structures. We found key differences in the conserved motifs in various *aaRS*s. For example, the class I motif 'KYSKS' in *P. falciparum TyrRS *(PF11_0181) and 'KMSKS' in MAL8P1.125 differs from 'KLGKS' of human mitochondrial *TyrRS *(2PID) and 'KMSSS' of human cytoplasmic (1N3L) respectively. Similarly, class I motif 'HIGH' has subtle sequences variations between *P. falciparum *and *H. sapiens TyrRSs *(Figure 7a, Table 3). Using the above procedures, we could generate structural models for several *Pf-aaRSs*. Stereo-chemical qualities of the generated protein models were assessed using PROCHECK (85-90% residues are in allowed regions of Ramachandran plot). The overall superimposed three-dimension models were visualized in CHIMERA and PYMOL (Figure 7b). Many sequence insertions were observed for *P. falciparum *enzymes when compared to their homologous [63]. Location of insertions in *P. falciparum TyrRS *between well-conserved secondary structures suggests ability of *TyrRS *anticodon binding core to accommodate larger sequence inserts with minimum disruption to the catalytic domain. Direct comparison of modeled *P. falciparum aaRS*s with human *aaRS*s revealed several other important structural differences. For example, numerous insertions are present in the loop regions linking various α-helices (α10 to α13) in anticodon binding domain of *P. falciparum TyrRSs *(PF11_0181 and MAL8p1.125) when compared to its human homologous (2PID and 1N3L) respectively. Structural differences between *TyrRS *(from *P. falciparum*) and human counterparts are summarized in Table 3 and shown in Figure 7c. These subtle structural changes that manifest as partial conservation of important motifs in *P. falciparum aaRSs *reflect evolutionary divergence, and may be useful for exploitation of parasite-specific features as drug targets.

**Table 3:** Structural differences between tyrosyl-tRNA synthetases from human &* P. falciparum*

	Hs-TyrRS (2PID)*	*Pf-TyrRS(PF11_0181)^@^*	*Hs-TyrRS (1N3L)^!^*	*Pf-TyrRS (MAL8P1.125)^$^*
**Motif 1**	*HVGH*	*HLGN*	HAVY	*HIAQ*
**Motif 2**	*KLGKS*	*KYSKS*	KMSSS	*KMSKS*
**Residues involved in tyrosine and A73 recognition**	*Ser200*	*Arg229*	Gly46	*Gly67*
	*Gln202*	*Glu231*	Arg93	*Val116*
	*Met252*	*Gln279*	Ala340	*Lys370*
	*Ile274*	*Leu301*	Tyr341	*Val371*
**Insertions**	----	*Arg157-Glu175*		
	----	*Glu316-Leu321*	Met104-Ser107	*Glu142-Lys146*
	----	*Asn369-Lys422*		*Asn356-Lys360*

* *human *mitochondrial

^@ ^*P. falciparum *bacteria-like

^! ^*human *cytosolic

^$ ^*P. falciparum *human-like

## Conclusion

Aminoacyl-tRNA synthetases (*aaRS*s) link RNA with protein translation. Besides their key role in protein synthesis, *aaRS*s are also integral to various other cellular processes. *aaRS *enzymes have been the focus for antimicrobial drug discovery [64,65]. An example of clinical application of an *aaRS *inhibitor is provided by the antibiotic mupirocin (marketed as Bactroban), which selectively inactivates bacterial isoleucyl-tRNA synthetase [66]. Similarly, it has been shown that the broad-spectrum antifungal 5-fluoro-1,3-dihydro-1-hydroxy-2,1-benzoxaborole (AN2690) inhibits yeast cytoplasmic leucyl-tRNA synthetase by blocking editing site of the enzyme [67,68]. Therefore, presence of distinct or tinkered *P. falciparum aaRS *lends an opportunity for their exploitation as new drug targets against malaria. In this study, we have extensively analyzed *aaRS *sequences from *Plasmodium *species in terms of their mRNA/protein expression profiles, their cellular localization, their organelle targeting and their unique sequence/domain attributes. We have discovered several distinct *aaRS*s in *P. falciparum *with no clear human counterparts in terms of their overall domain structures. We have also highlighted deviations of some highly conserved sequence motifs and active site sequence clusters. Our analyses clearly show that a larger fraction of *P. falciparum *proteome is devoted to *aaRS *when compared with many other organisms. The phylogenetic data hint at evolutionary closeness of some *Pf-aaRSs *to bacteria and plants - this further supports the fact of secondary endosymbiosis in this apicomplexan. We hope that our in-depth phylogenetic, protein targeting, domain architecture, protein expression profiling and homology modeling data on *Pf-aaRSs *can be used as a platform for experimental studies of this important protein family in malaria parasites.

## Methods

### Sequence extraction

The *P. falciparum *genome database PlasmoDB Release 5.4 was used for the present analyses. Sequence sets of all the *aaRS*s from other organisms includes *P. berghei, P. chabaudi, P. falciparum, P. knowlesi, P. yoelii, P. vivax, H. sapiens, M. tuberculosis, D. discoidium, M. jannaschii, R. norvegicus, C. parvum, B. bovis, S. cerevisiae, D. melanogaster, Y. pestis, T. aquaticus, S. pneumoniae, S. entrica, E. coli, A. thaliana, A. pisum, A. salmonicida, B. cereus, B. thuringiensis, B. afzelii, B. burgdorferi, B. garinii, B. valaisiana, Bradyrhizobium, B. pennsylvanicus, C. acidaminovorans, H. defensa, C. taiwanensis, E. fergusonii, F. bacterium, F. novicida, F. tularensis, F. alni, G. tenuistipitata, H. arsenicoxydans, A. cellulolyticus, A. chlorophenolicus, A. ferrooxidans, Algoriphagus, A. muciniphila, Anaeromyxobacter, A. thermophilum, B. ambifaria, B. indica, B. mycoides, B. taurus, B. tribocorum, C. atlanticus, Caulobacter, C. aurantiacus, C. cellulolyticum, Citrobacter, C. pinensis, C. Ruthia, Cyanothece, D. desulfuricans, D. hafniense, Diaphorobacter, D. shibae, D. turgidum, E. cuniculi, E. lenta, E. ruminantium, Exiguobacterium, G. diazotrophicus, Geobacillus, M. maris, N. multipartita, Nocardioides, O. terrae, P. abelii, P. atlantica, P. denitrificans, P. ingrahamii, P. lavamentivorans, R. castenholzii, S. arenicola, S. fumaroxidans, X. autotrophicus, V. vadensis, V. paradoxus, T. whipplei, T. auensis, S. stellata, Ch. parvum, S. heliotrinireducens, Silicibacter, S. putrefaciens, S. usitatus, Thauera, X. laevis, Theileria annulata, Vibrio fischeri, W. succinogenes, X. tropicalis, Zeamays*. Additional sequences were obtained based on sequence similarity via NCBI BLAST [69] and ENSEMBL [70] databases. Known sequence motifs of *aaRS*s have been used as templates to retrieve sequences of *aaRS *from other organisms. Some *aaRS *sequences were manually annotated based on the presence of signature motifs. Protein domains and motifs in the predicted *aaRS*s were identified using following programs - Superfamily [71], SMART [72] and MotifScan available at expasy web server. The following databases - Pfam [73], TIGR, PIR, EBI and PlasmoDB were also extensively used. Hidden Markov Model (HMM) for each of the 20 *aaRS *were constructed by the software package Sequence Alignment and Modeling System version 2.2.1 (SAM) [74] exploiting sequences in the *aaRS *database [75]. HMM profiles were then used to carry out database search *vs P. falciparum *proteins. A score was assigned to each protein by calculating the probability that the corresponding sequence is generated by the HMM model, hence for each database search a score distribution was obtained. The score distributions were normalized and 4 ranges of values were considered to identify aa*RS *(c > 5, 10 < c < 20, 20 < c < 50, c < 50).

### Expression and Localization

The prediction of signal sequences for cellular localization in *P. falciparum *was performed using various available online web-servers - MITOPROT [76], PredictNLS [77] and PATS [78] for mitochondria, nucleus and apicoplast respectively. PEXEL motif prediction was been carried out by querying PlasmoDB. To identify specific gene expression profiles, we have combined information from different data sets. For the spotted oligonucleotide array data, only half of the 48 time points of the intra-erythrocytic cycle are shown for simplicity, and ratios (versus a common reference) were log_2_-transformed prior to cluster analysis. For the photolithography data, CEL files were downloaded from website and transferred into Bioconductor package for analysis using a robust multi-array averaging algorithm (RMA) for background adjustment and quantiles normalization [79]. Genes whose expression level was less than 10 (too close to background) or the logP was greater than -0.5 (too few probes per gene) were removed from dataset. Total intensity values for each time point were converted to mean-centered ratios by dividing the total intensity by the average intensity for that gene across all experimental conditions and were then log_2_-transformed prior to clustering. These data manipulations were necessary because the oligo-nucleotide array data was collected as the intensity ratio between the experimental sample and a common reference, while the photolithography data was collected as the total signal intensity at each spot. Gene expression patterns where the minimum percentage of existing values was less than 80% were eliminated from rest of the analysis. The remaining missing values were replaced by using the KNN-imputation method [80].

### Phylogenetic analysis

To explore the evolutionary relationships amongst *aaRSs *phylogenetic analyses were performed for each *P. falciparum aaRS *on an expanded set of 102 sequences. Multiple sequence alignments of these sequences were obtained from CLUSTALW with default parameters (performed locally) in PHYLIP format [81]. These MSAs were used as seed sequences to run PHYML_v2.4.4 using Jones-Taylor-Thornton (JTT) model [82]. The resulting file was further used in MEGA4.2 for visualization of trees [83].

### Model Building and Validation

We used Sali's Modeller8v2 [84] tool for building various *P. falciparum aaRS*s models. The stereo-chemical quality of modeled proteins was verified by PROCHECK [85]. Structural mapping of active site residues and other motifs was performed using CHIMERA [86] and PYMOL [87].

## Authors' contributions

TKB, CK and SK carried out the computational experiments and data analysis and wrote the paper; MAJ and VS contributed to the manuscript writing; DS and EP carried out HMM construction and database search by HMM; FS performed analysis of transcriptomic and proteomic data; AS designed the study and supervised the work. All authors have read and approved the final manuscript.

## Supplementary Material

Additional file 1 **List of *Pf-aaRSs *categorized into class I, class II, and related proteins**. Gene ID, gene location, description of product and its length are given. Click here for file

Additional file 2 **Phylogenetic trees of *aaRSs *from *P. falciparum***. The evolutionary tree was constructed by the method PHYML using the MEGA 4.0. *P. falciparum *aaRSs are labeled green triangles. 102 species considered for the evolutionary analysis are taken from the three domains of life *viz. P. berghei, P. chabaudi, P. falciparum, P. knowlesi, P. yoelii, P. vivax, H. sapiens, M. tuberculosis, D. discoidium, M. jannaschii, R. norvegicus, C. parvum, B. bovis, S. cerevisiae, D. melanogaster, Y. pestis, T. aquaticus, S. pneumoniae, S. entrica, E. coli, A. thaliana, A. pisum, A. salmonicida, B. cereus, B. thuringiensis, B. afzelii, B. burgdorferi, B. garinii, B. valaisiana, Bradyrhizobium, B. pennsylvanicus, C. acidaminovorans, H. defensa, C. taiwanensis, E. fergusonii, F. bacterium, F. novicida, F. tularensis, F. alni, G. tenuistipitata, H. arsenicoxydans, A. cellulolyticus, A. chlorophenolicus, A. ferrooxidans, Algoriphagus, A. muciniphila, Anaeromyxobacter, A. thermophilum, B. ambifaria, B. indica, B. mycoides, B. taurus, B. tribocorum, C. atlanticus, Caulobacter, C. aurantiacus, C. cellulolyticum, Citrobacter, C. pinensis, C. Ruthia, Cyanothece, D. desulfuricans, D. hafniense, Diaphorobacter, D. shibae, D. turgidum, E. cuniculi, E. lenta, E. ruminantium, Exiguobacterium, G. diazotrophicus, Geobacillus, M. maris, N. multipartita, Nocardioides, O. terrae, P. abelii, P. atlantica, P. denitrificans, P. ingrahamii, P. lavamentivorans, R. castenholzii, S. arenicola, S. fumaroxidans, X. autotrophicus, V. vadensis, V. paradoxus, T. whipplei, T. auensis, S. stellata, Ch. parvum, S. heliotrinireducens, Silicibacter, S. putrefaciens, S. usitatus, Thauera, X. laevis, Theileria annulata, Vibrio fischeri, W. succinogenes, X. tropicalis, Zeamays*. Click here for file
