# Supplementary material for: A genomic glimpse of aminoacyl-tRNA synthetases in malaria parasite Plasmodium falciparum
Source: BMC Genomics. 2009 Dec 31;10:644. doi: 10.1186/1471-2164-10-644 (PMC2813244; doi:10.1186/1471-2164-10-644)
Supplement: Additional file 1 — List of Pf-aaRSs categorized into class I, class II, and related proteins. Gene ID, gene location, description of product and its length are given. [file 1471-2164-10-644-S1.PDF]

Additional file 1. List of *Pf-aaRS categorized into class I , class II and related proteins*

[Gene]	[Genomic Location]	[Product Description]	[Protein Length]
**Class I**			
PFI0680c	Pf3D7_9: 594,263 - 597,225 (-)	arginyl-tRNA synthetase	943
PFL0900c	Pf3D7_12: 732,167 - 734,952 (-)	arginyl-tRNA synthetase, putative	590
PF10_0149	Pf3D7_10: 614,871 - 616,795 (-)	cysteine-tRNA ligase, putative	593
PF13_0257	Pf3D7_13: 1,968,221 - 1,970,812 (-)	glutamate-tRNA ligase	863
MAL13P1.281	Pf3D7_13: 2,267,870 - 2,270,416 (+)	glutamate-tRNA ligase, putative	574
PF13_0170	Pf3D7_13: 1,319,287 - 1,322,043 (-)	glutaminyl-tRNA synthetase, putative	918
PFL1210w	Pf3D7_12: 1,018,497 - 1,023,999 (+)	isoleucine-tRNA syntheyase	1696
PF13_0179	Pf3D7_13: 1,350,240 - 1,354,058 (-)	isoleucine-tRNA ligase, putative	1272
PF08_0011	Pf3D7_8: 1,217,343 - 1,221,788 (+)	leucine-tRNA ligase	1481
PFF1095w	Pf3D7_6: 919,683 - 924,026 (+)	leucyl-tRNA synthetase, cytoplasmic	1447
PF10_0340	Pf3D7_10: 1,383,447 - 1,386,116 (+)	methionine-tRNA ligase, putative	889
PF10_0053	Pf3D7_10: 225,457 - 227,706 (-)	methionyl-tRNA ligase, putative	749
PF13_0205	Pf3D7_13: 1,488,257 - 1,490,458 (-)	tryptophan-tRNA ligase, putative	632
PFL2485c	Pf3D7_12: 2,106,329 - 2,108,278 (-)	tryptophanyl-tRNA synthetase, putative	570
PF11_0181	Pf3D7_11: 665,647 - 667,721 (-)	tyrosine-tRNA ligase, putative	613
MAL8P1.125	Pf3D7_8: 403,717 - 405,263 (+)	tyrosyl-tRNA synthetase, putative	373
PF14_0589	Pf3D7_14: 2,514,608 - 2,517,880 (+)	valine-tRNA ligase, putative	1090
PFC0470w	Pf3D7_3: 471,464 - 475,762 (+)	valine-tRNA ligase, putative	1367
**Class II**			
PF13_0354	Pf3D7_13: 2,691,989 - 2,696,215 (+)	alanine-tRNA ligase, putative	1408
PFE0475w	Pf3D7_5: 403,342 - 405,642 (+)	asparagine-t RNA ligase, putative	722
PFB0525w	Pf3D7_2: 475,242 - 477,074 (+)	asparagine-tRNA ligase, putative	610
PFE0715w	Pf3D7_5: 600,045 - 603,431 (+)	aspartyl-tRNA ligase, putative	1128
PFA0145c	Pf3D7_1: 129,194 - 131,074 (-)	aspartyl-tRNA synthetase	626
PF14_0198	Pf3D7_14: 846,533 - 849,172 (+)	glycine-tRNA ligase, putative	879
PF14_0428	Pf3D7_14: 1,851,599 - 1,854,997 (-)	histidine-tRNA ligase, putative	1132
PFI1645c	Pf3D7_9: 1,340,054 - 1,341,982 (-)	histidyl-tRNA synthetase	642
PF14_0166	Pf3D7_14: 682,777 - 684,801 (+)	lysine-tRNA ligase, putative	674
PF13_0262	Pf3D7_13: 2,002,293 - 2,004,281 (-)	lysine-tRNA ligase	583
PF11_0051	Pf3D7_11: 175,609 - 177,779 (-)	phenylalanine-tRNA ligase beta, putative	623
PFL1540c	Pf3D7_12: 1,325,740 - 1,327,329 (-)	phenylalanyl-tRNA synthetase (likely monomeric)	529
PFA0480w	Pf3D7_1: 381,851 - 383,578 (+)	phenylalanyl-tRNA synthetase alpha chain	575
PFF0180w	Pf3D7_6: 151,052 - 152,383 (+)	phenylalanyl-tRNA synthetase (likely monomeric)	443
PFI1240c	Pf3D7_9: 1,019,154 - 1,020,893 (-)	prolyl-tRNA synthase, putative	579
PFL0770w	Pf3D7_12: 644,532 - 646,388 (+)	seryl-tRNA synthetase, putative	618
PF07_0073	Pf3D7_7: 820,170 - 821,789 (-)	seryl-tRNA synthetase, putative	539
PF11_0270	Pf3D7_11: 1,014,333 - 1,017,374 (-)	threonine-tRNA ligase, putative	1013
PFL0670c*	Pf3D7_12: 590,187 - 592,427 (-)	bifunctional (prolyl) aminoacyl-tRNA synthetase	746
**Related proteins**			
PF14_0401	Pf3D7_14: 1,712,569 - 1,714,008	EMAP-II-like cytokine	402
PFF1395c	Pf3D7_ 6: 1,185,997 to 1,188,579	glutamyl-tRNA(Gln) amidotransferase subunit B	860
PFD0780w	Pf3D7_ 4: 713,512 to 715,992	glutamyl-tRNA(Gln) amidotransferase subunit A	826
PFC0395w	Pf3D7_ 3: 399,607-402,114(+)	asparagine (amido) synthetase putative	610
PF14_0423	Pf3D7_ 14: 1,833,725 to 1,838,541 (-)	Ser/Thr protein kinase, putative	1558

***PFL0670c is likely to be prolyl-tRNA synthetase**
