# Supplementary figures and images for: A genomic glimpse of aminoacyl-tRNA synthetases in malaria parasite Plasmodium falciparum
Source: BMC Genomics. 2009 Dec 31;10:644. doi: 10.1186/1471-2164-10-644 (PMC2813244; doi:10.1186/1471-2164-10-644)

ALA

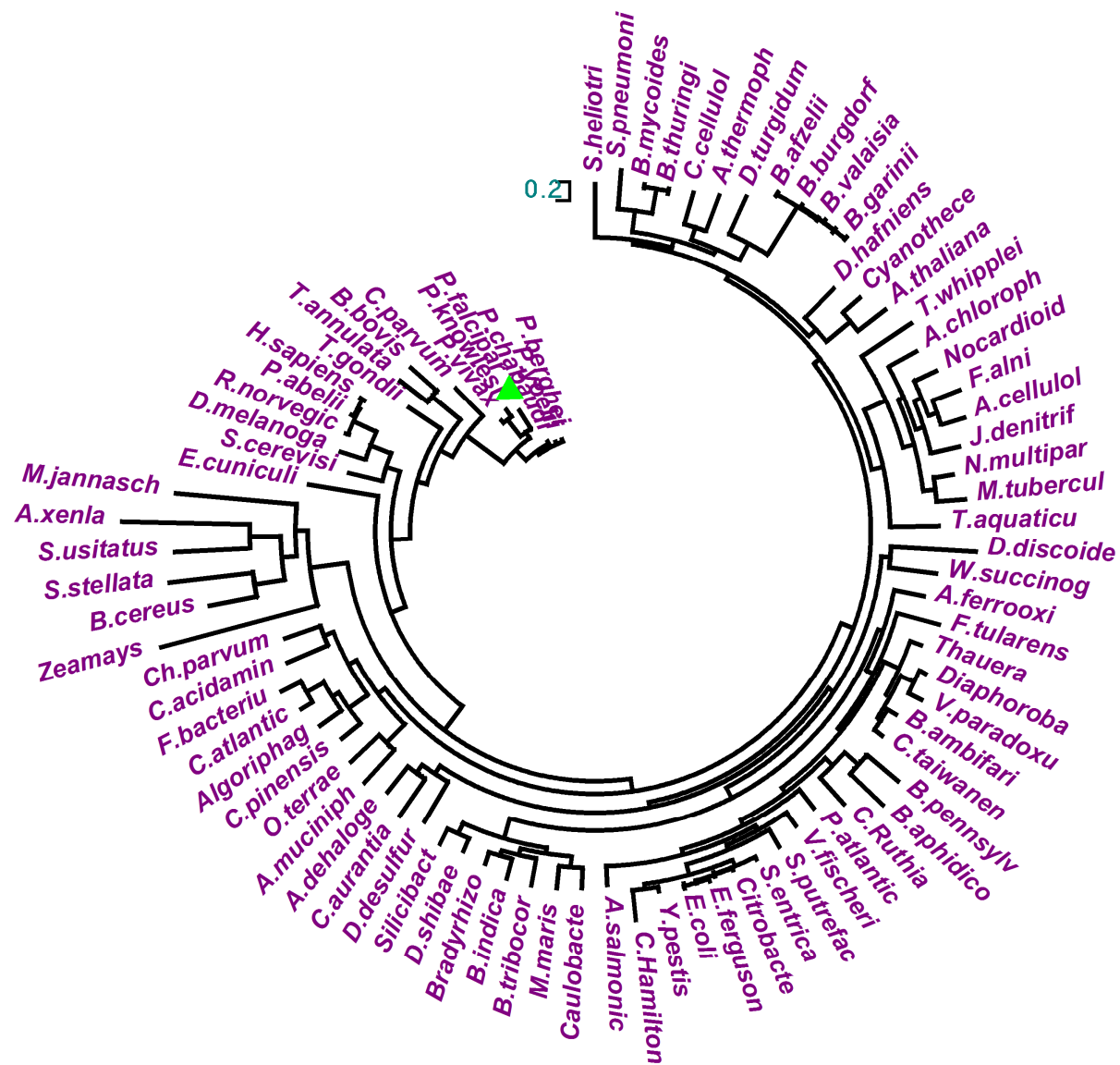

ARG

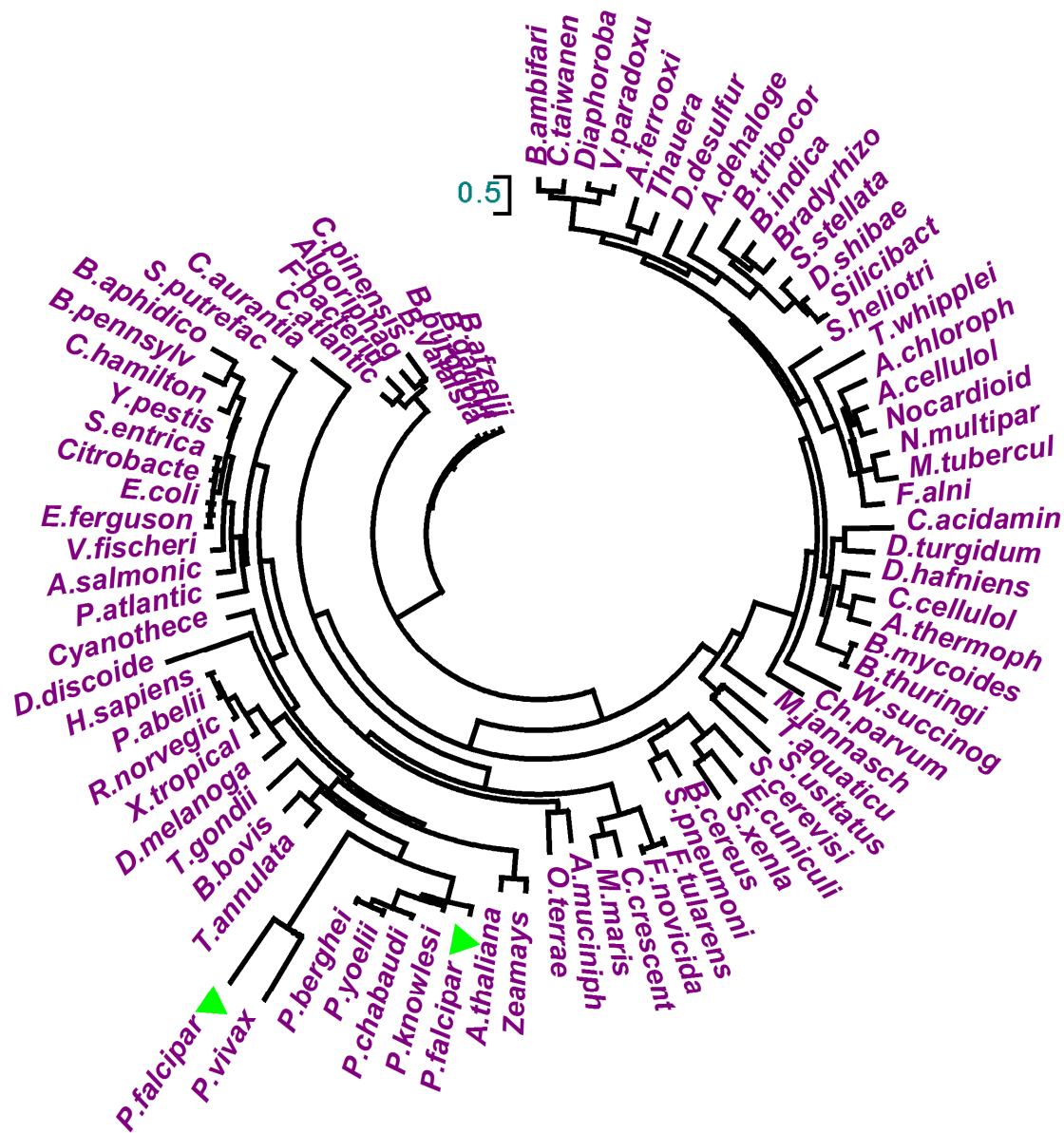

ASN

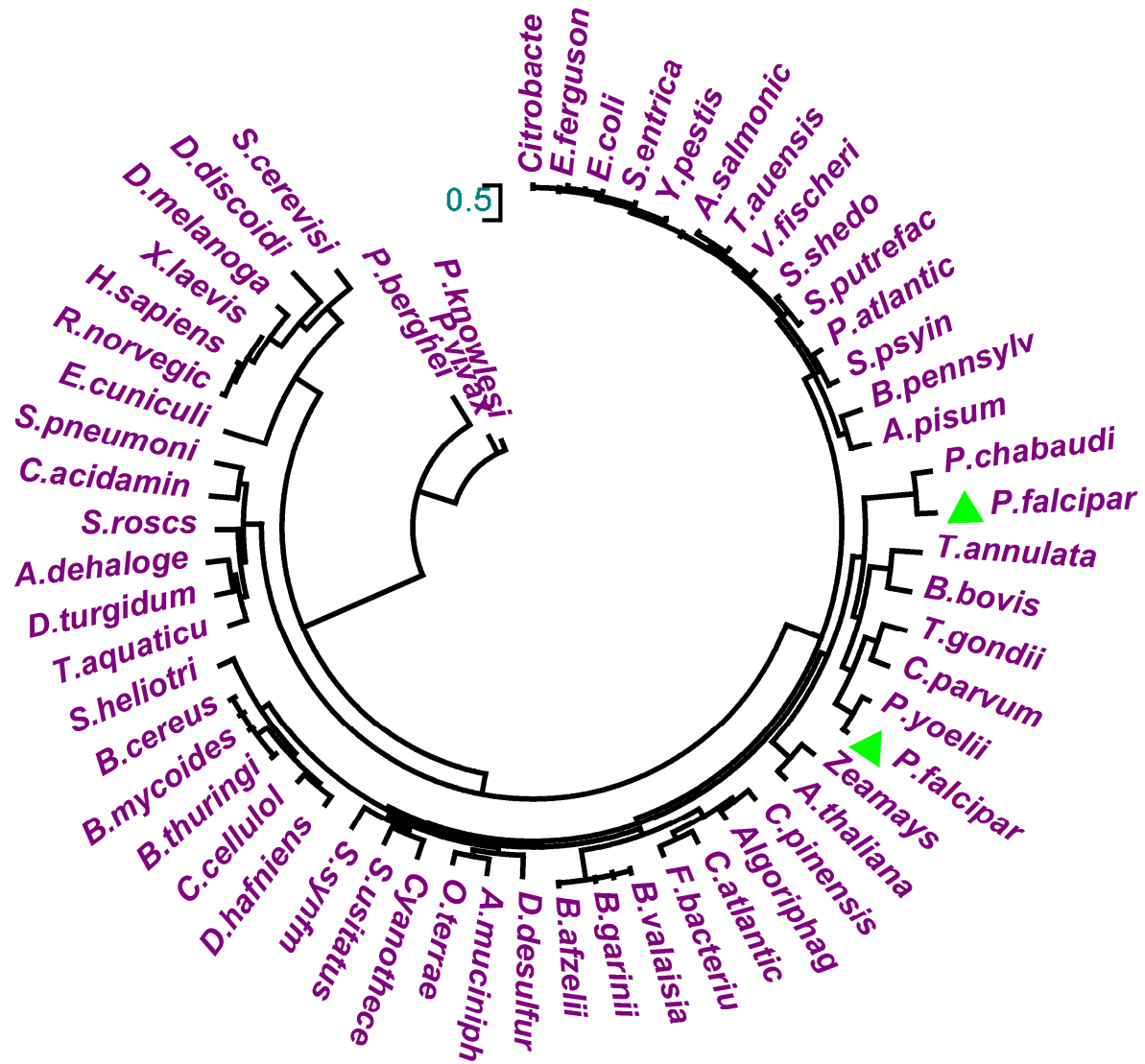

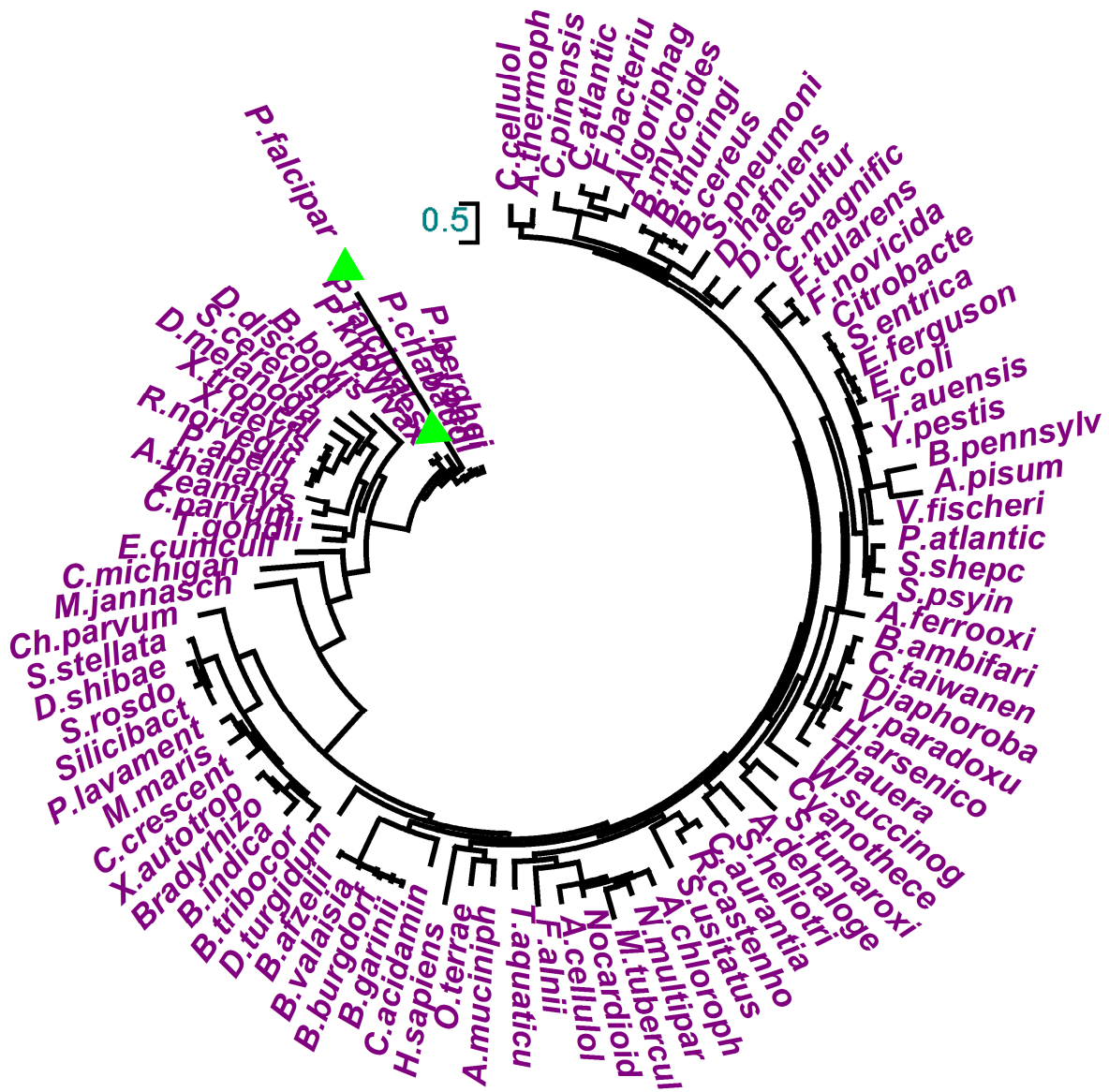

CYS

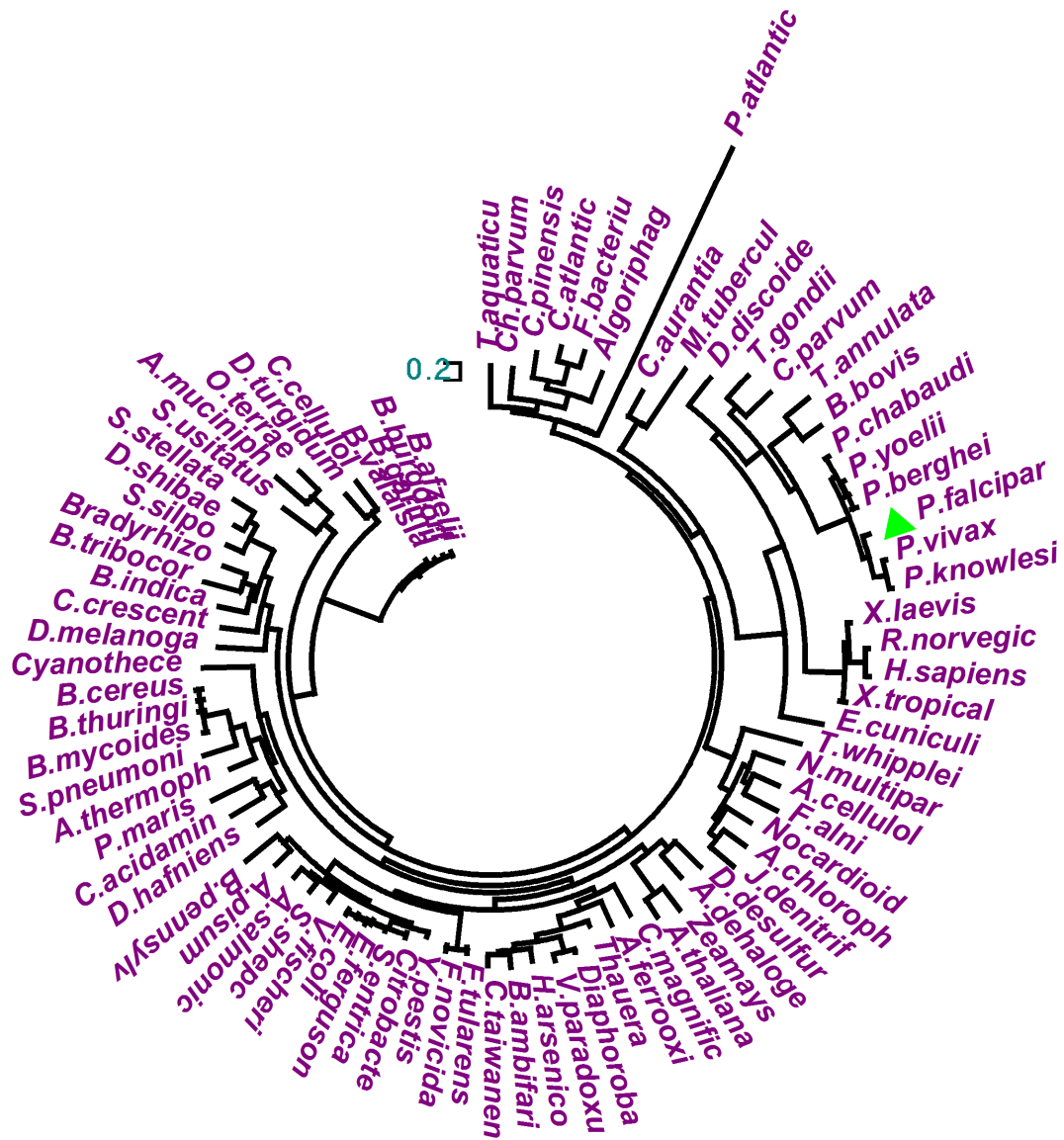

GLN

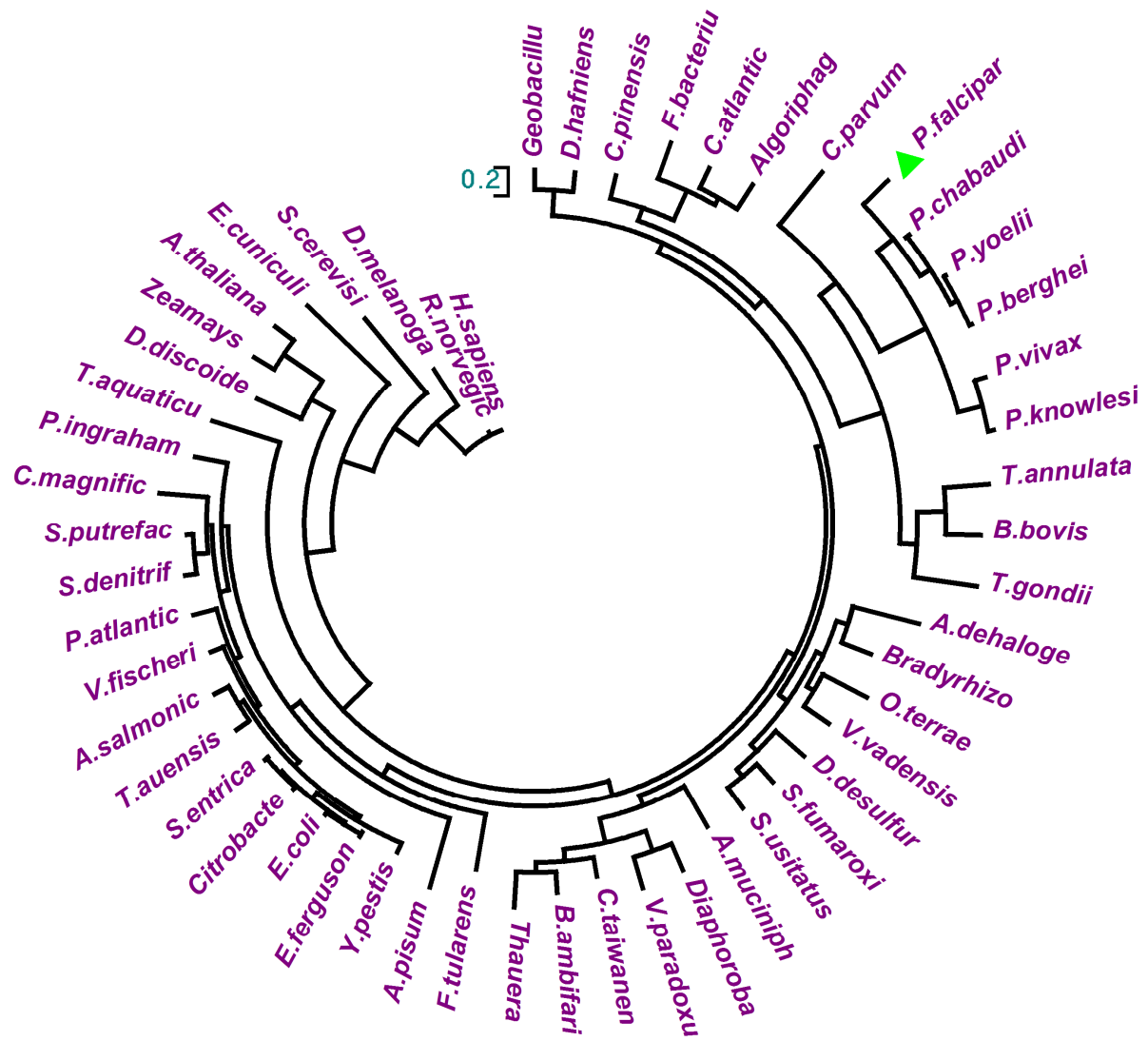

GLU

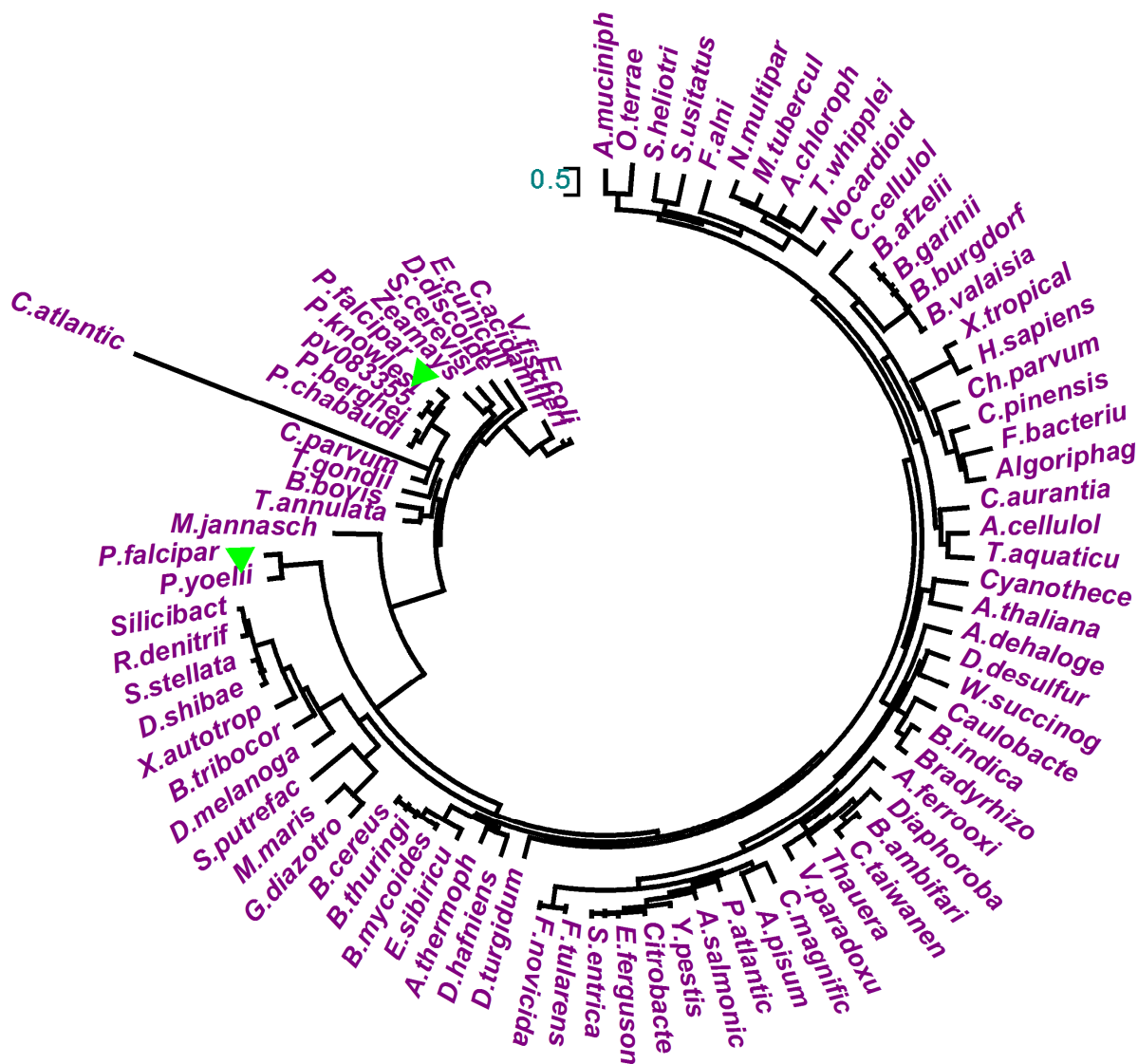

GLY

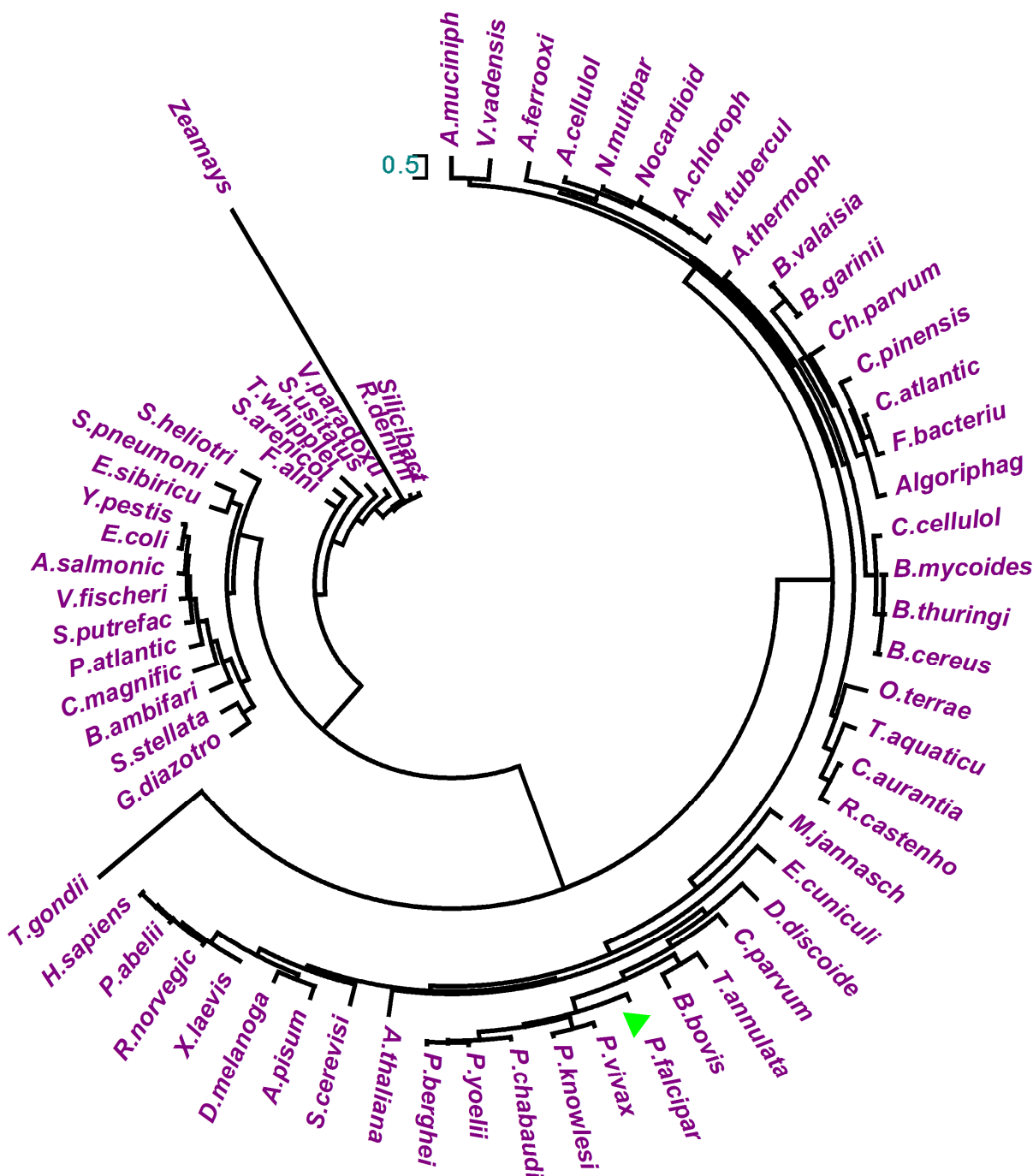

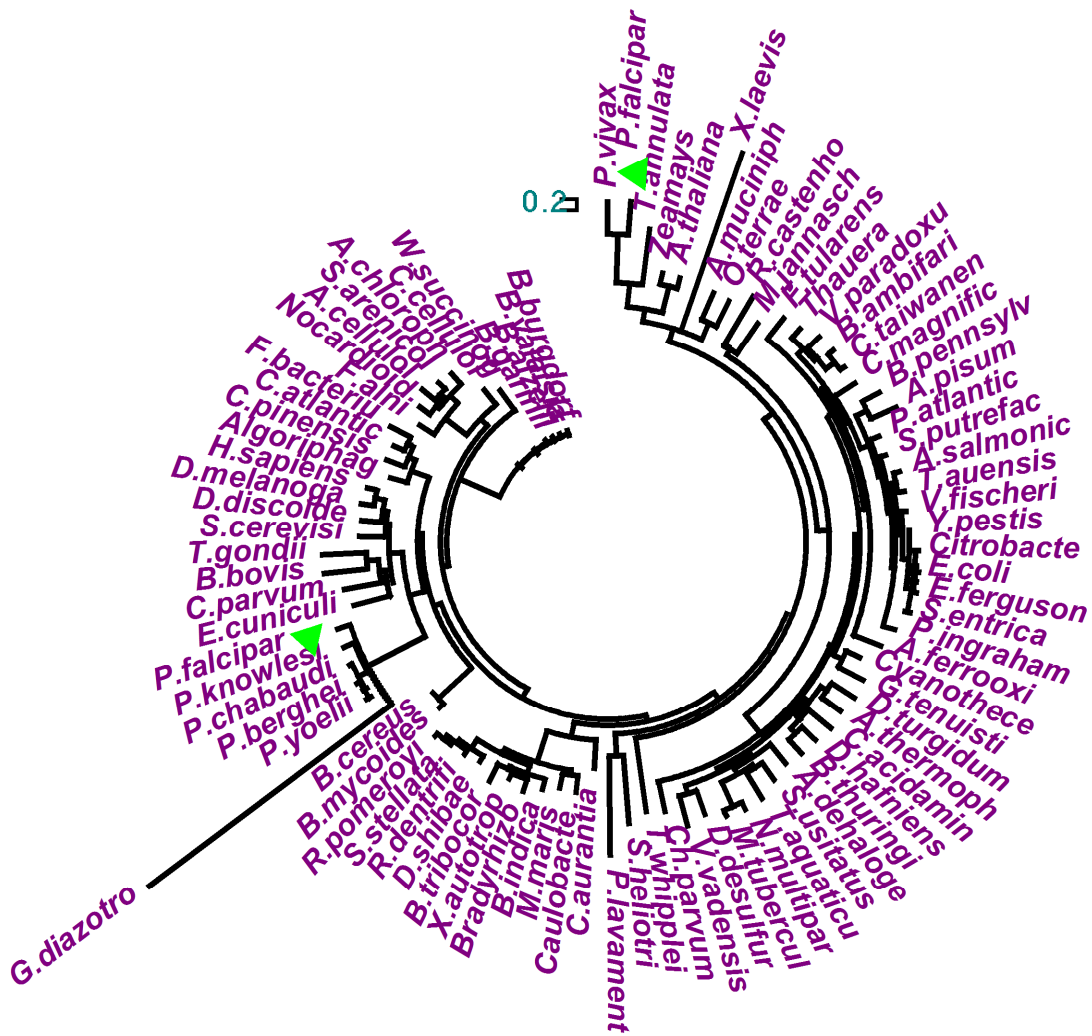

ILE

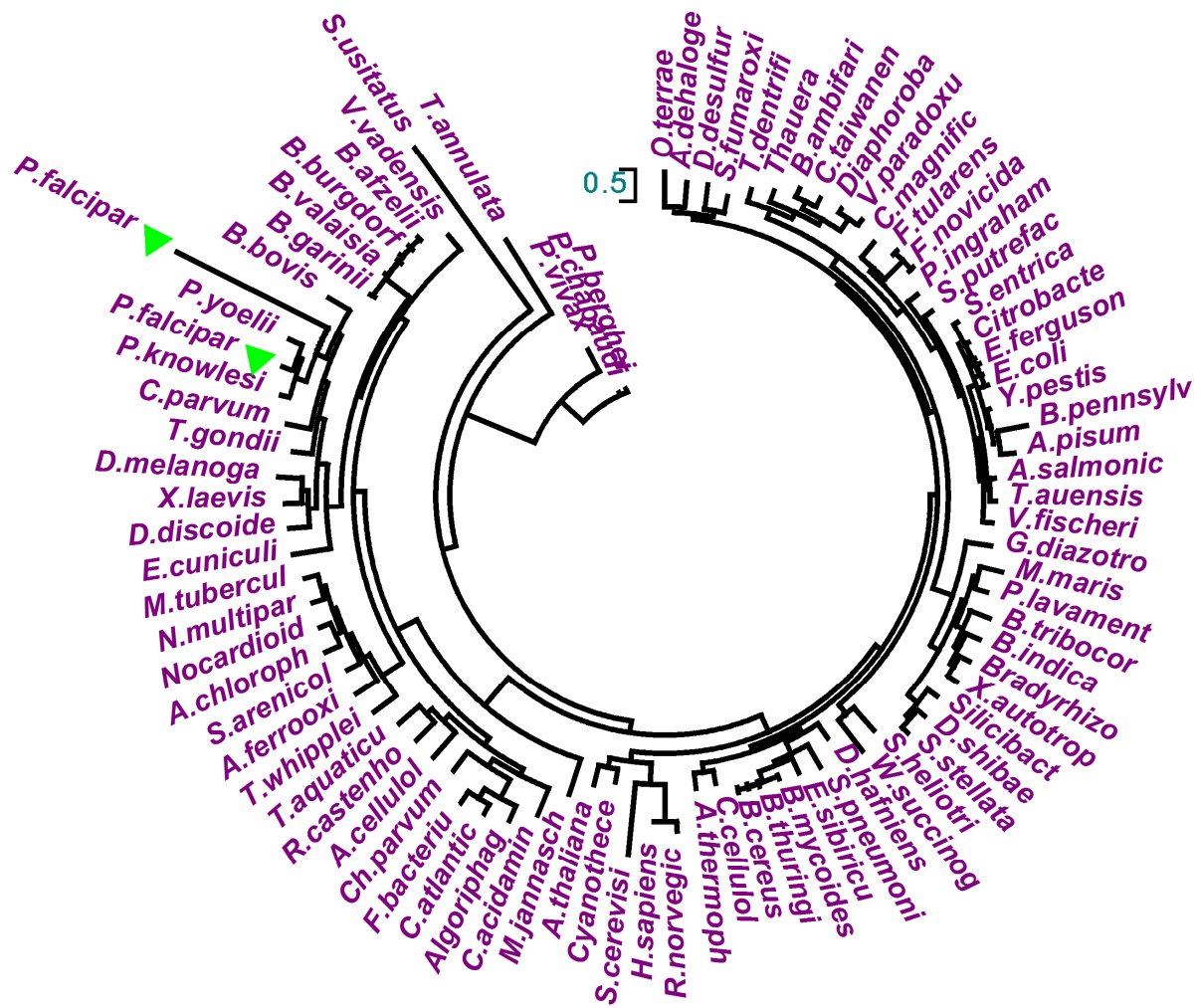

LEU

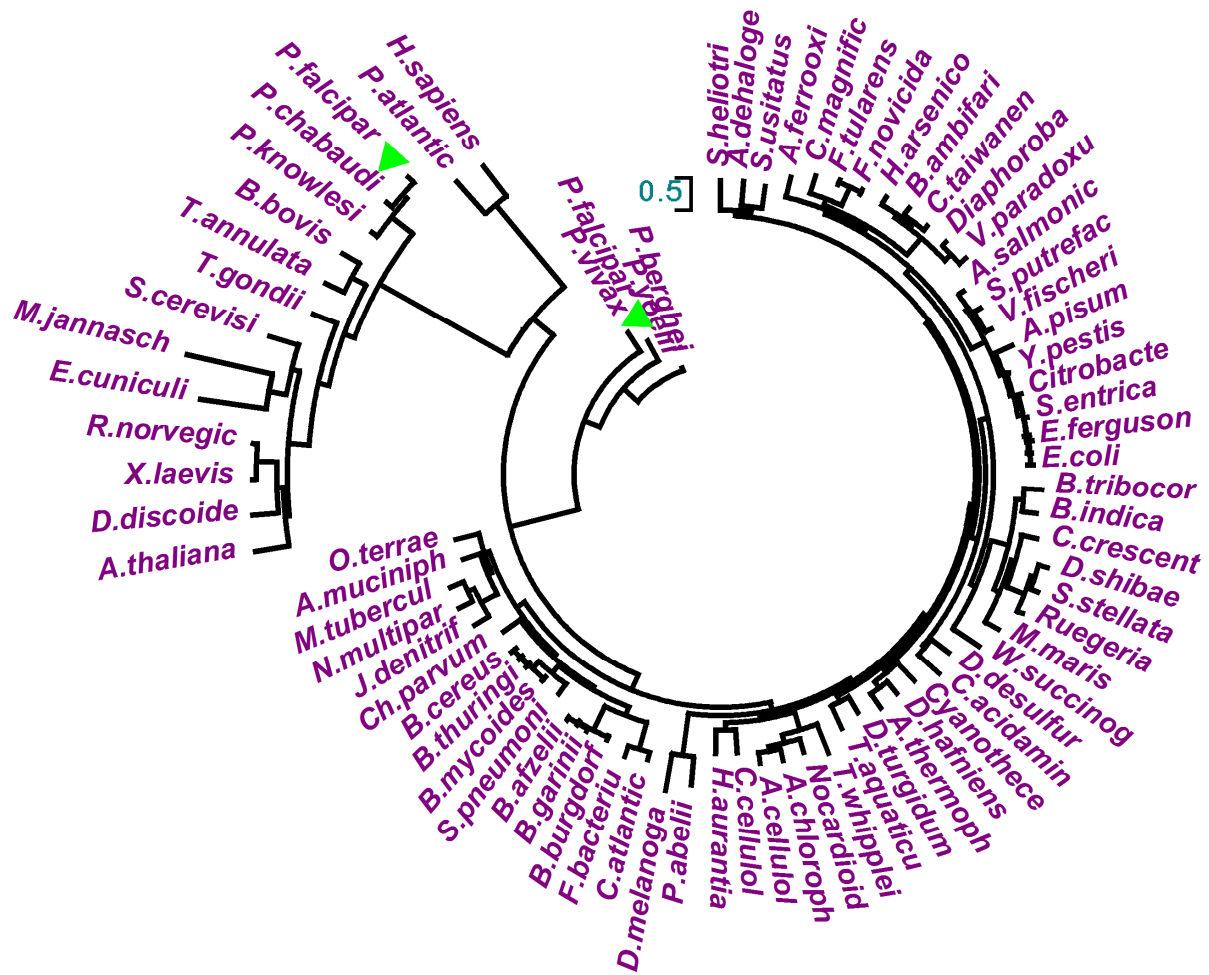

LYS

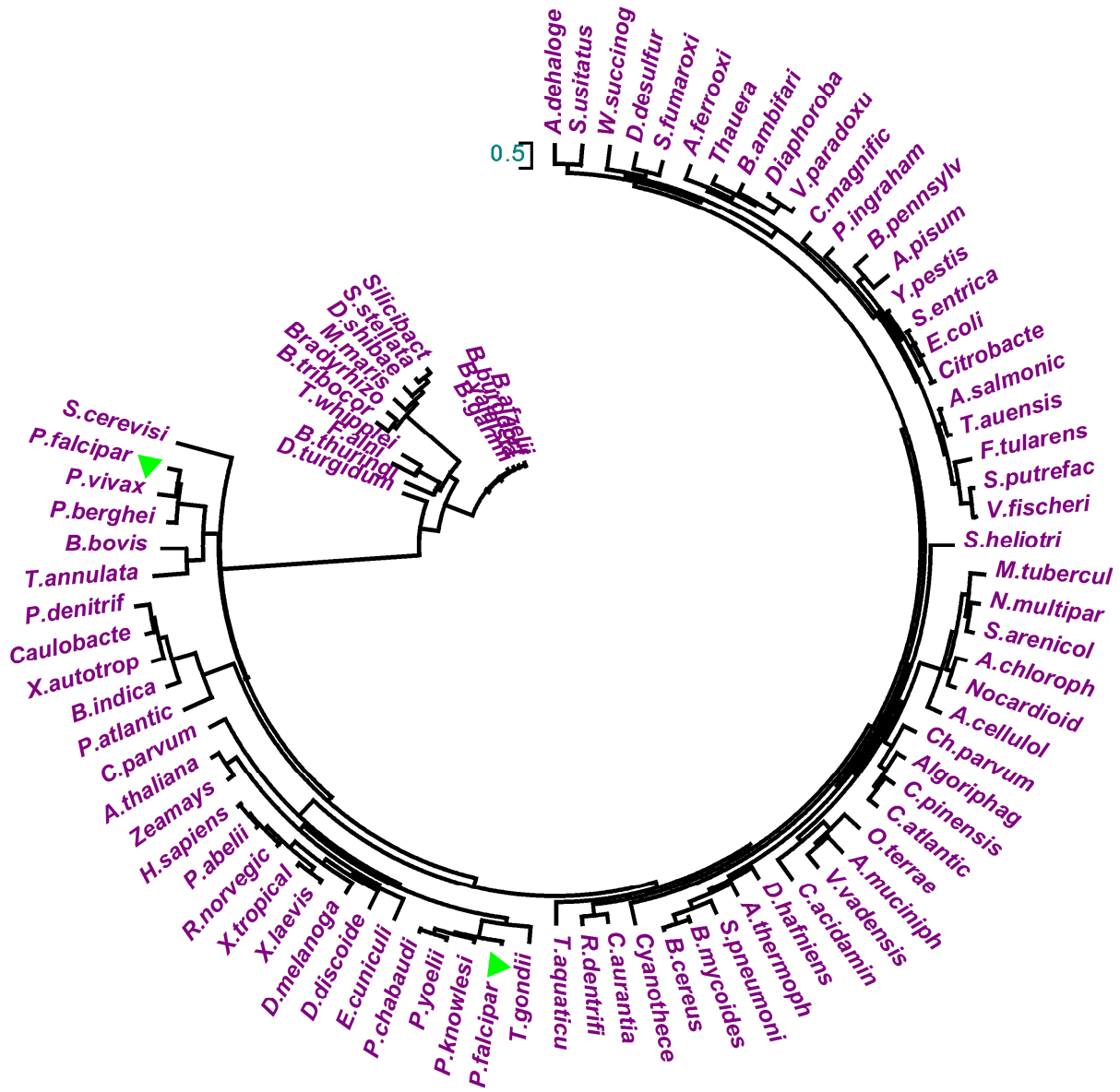

MET

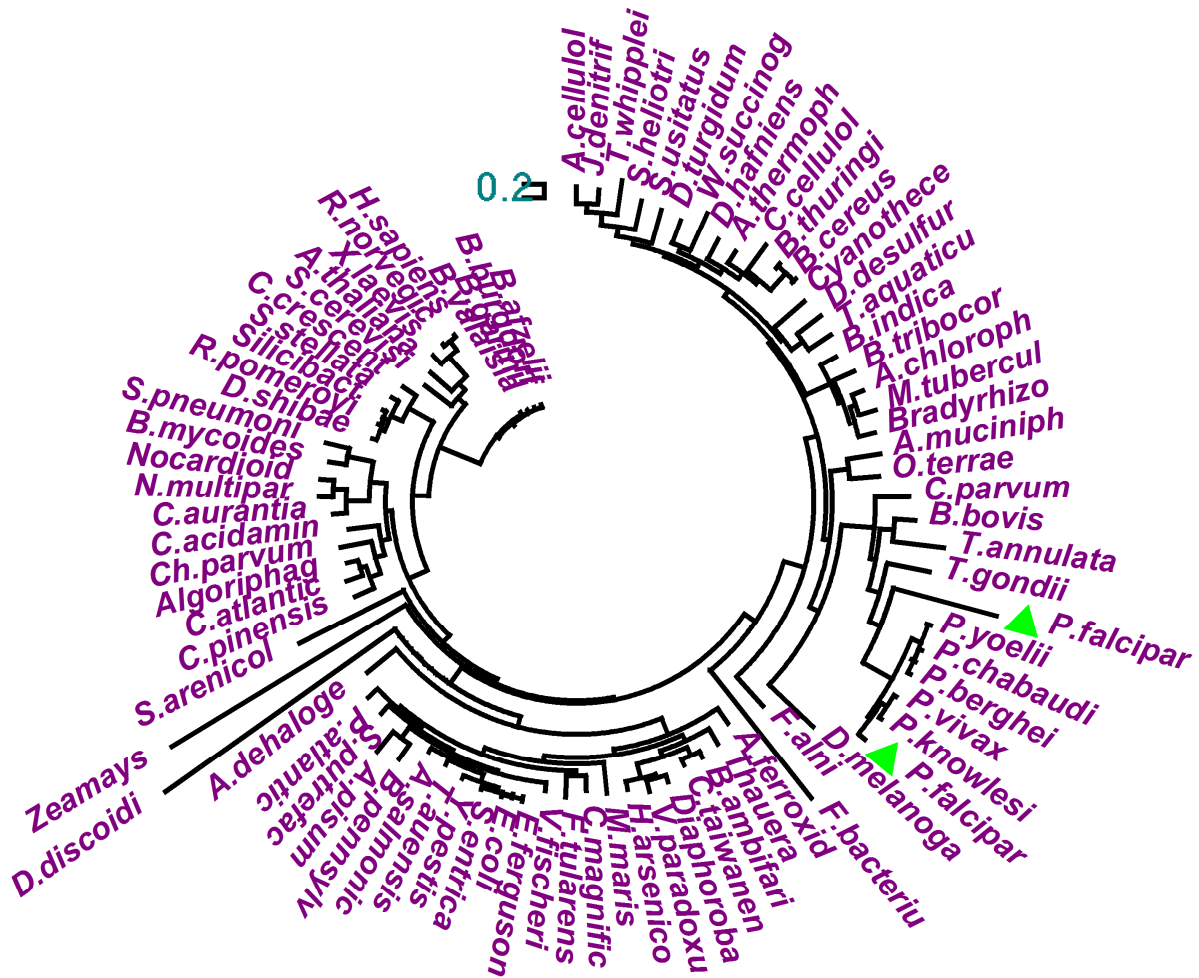

PHE ALPHA

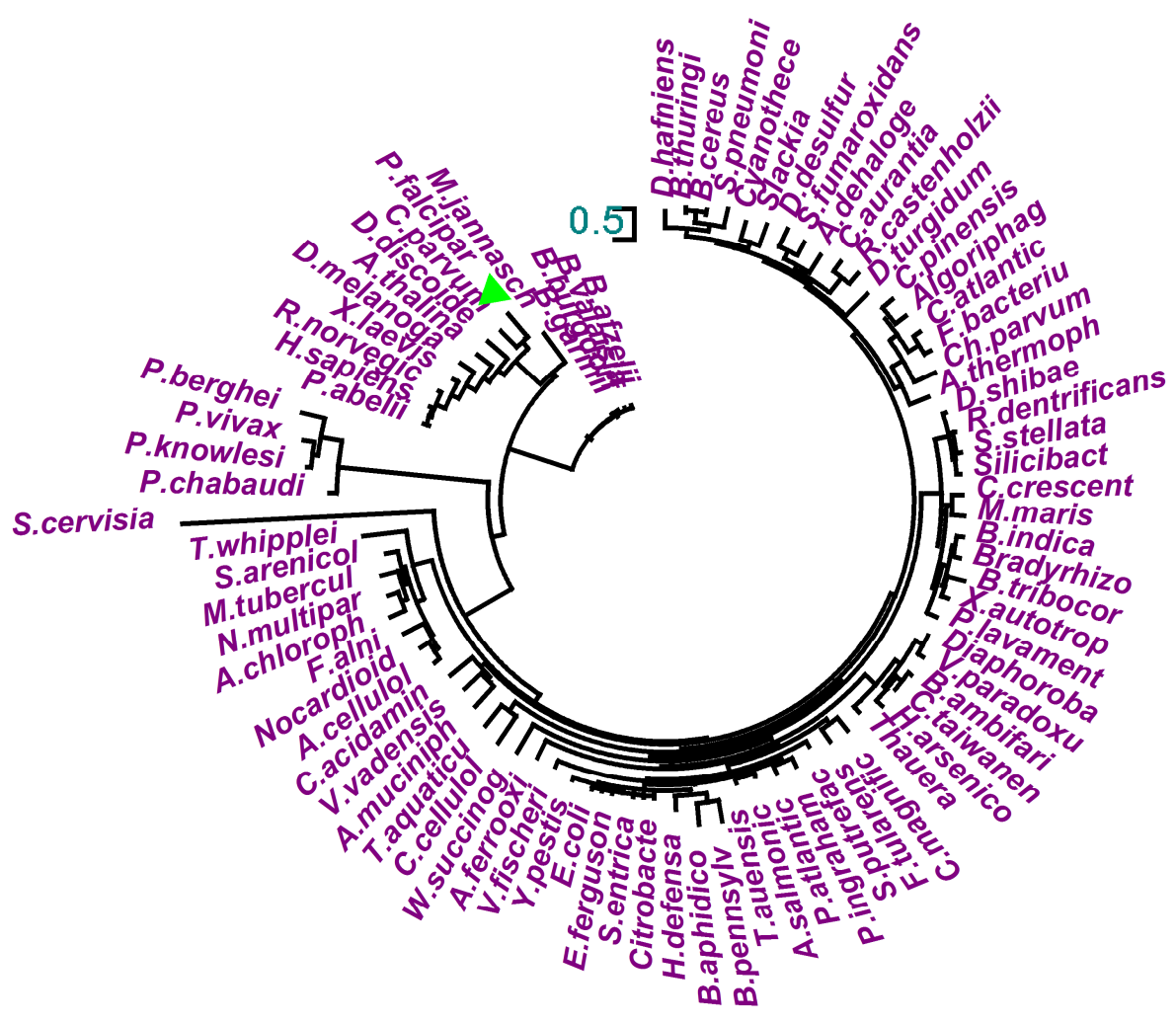

PHE BETA

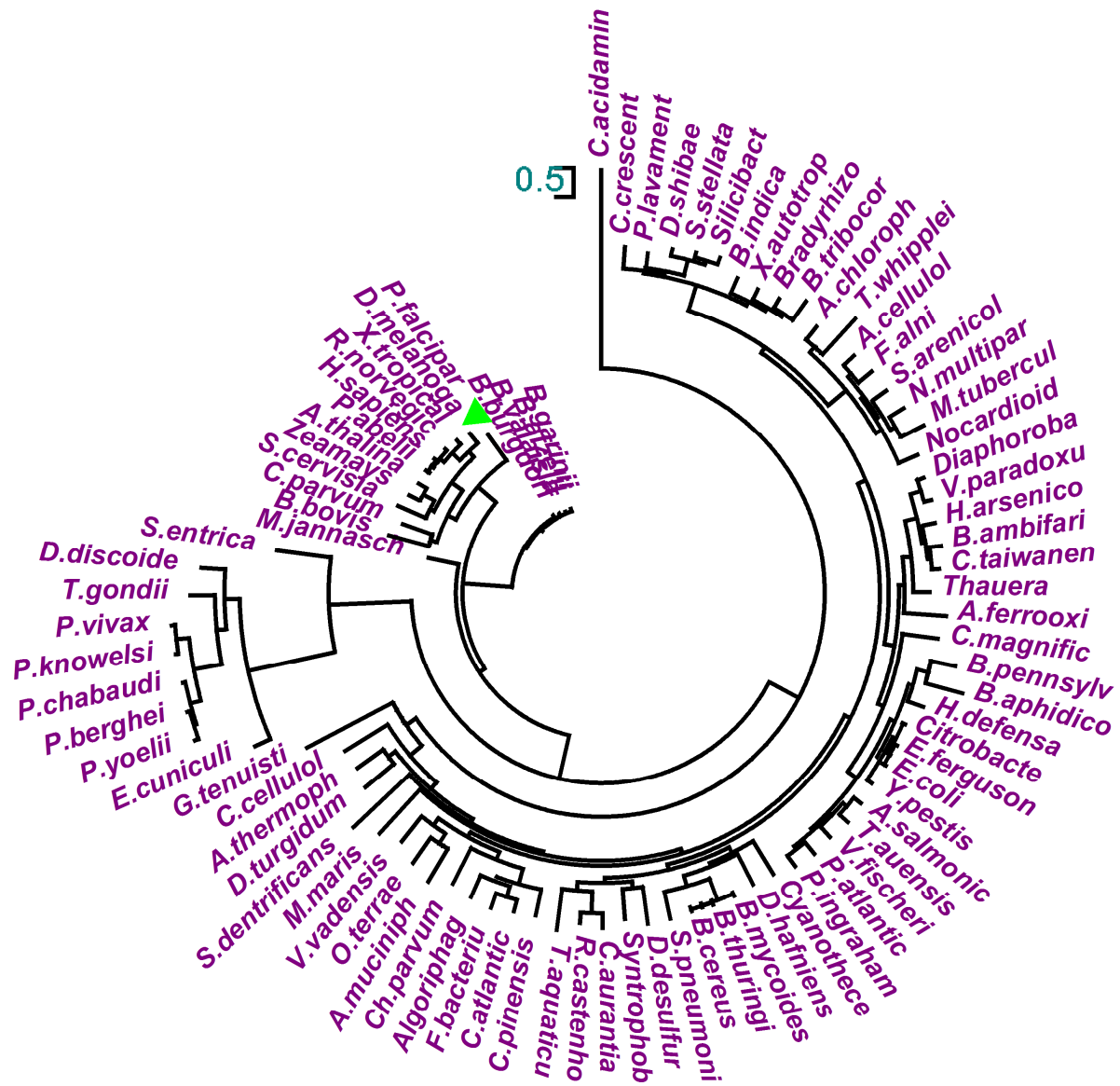

PRO

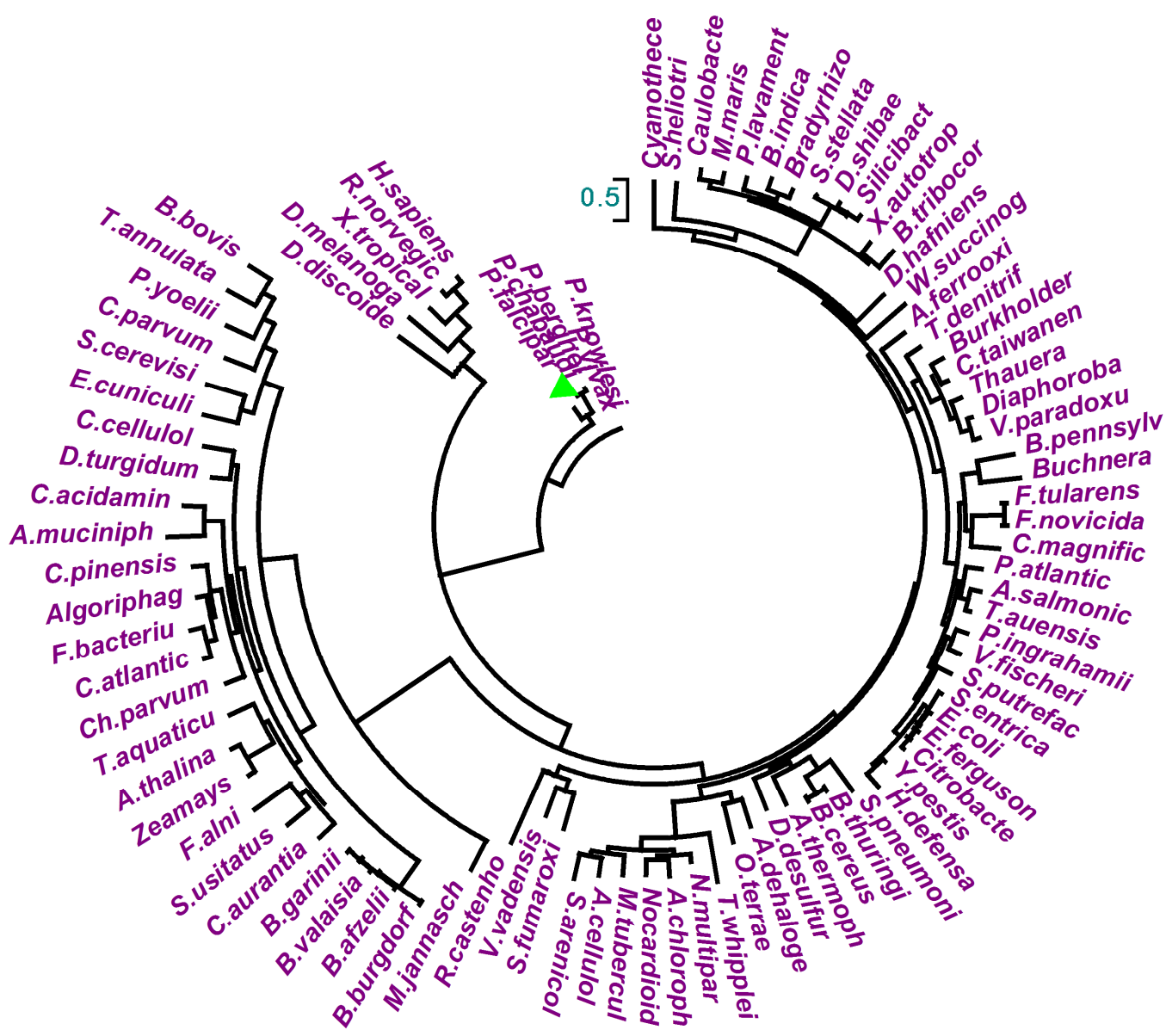

SER

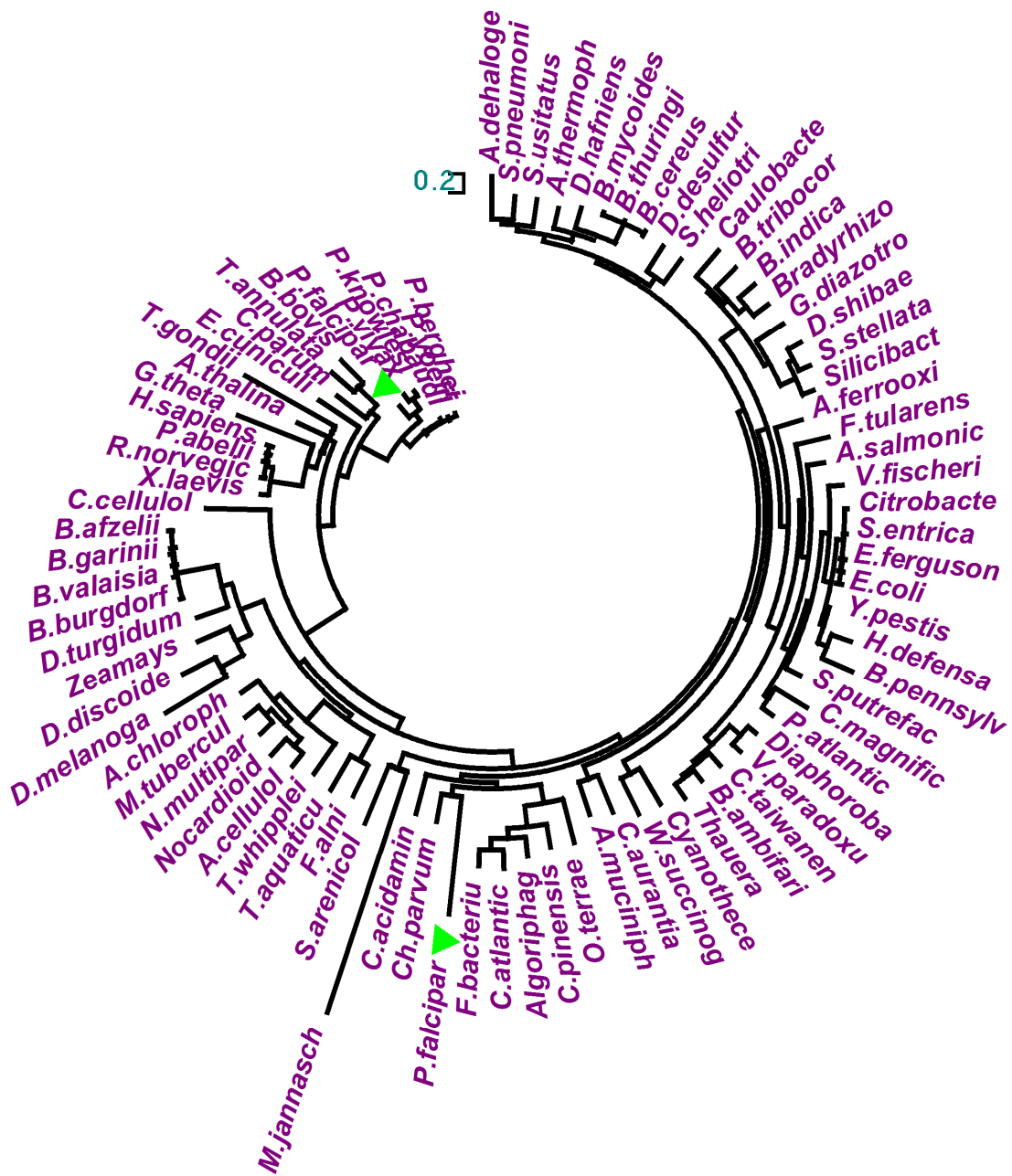

THR

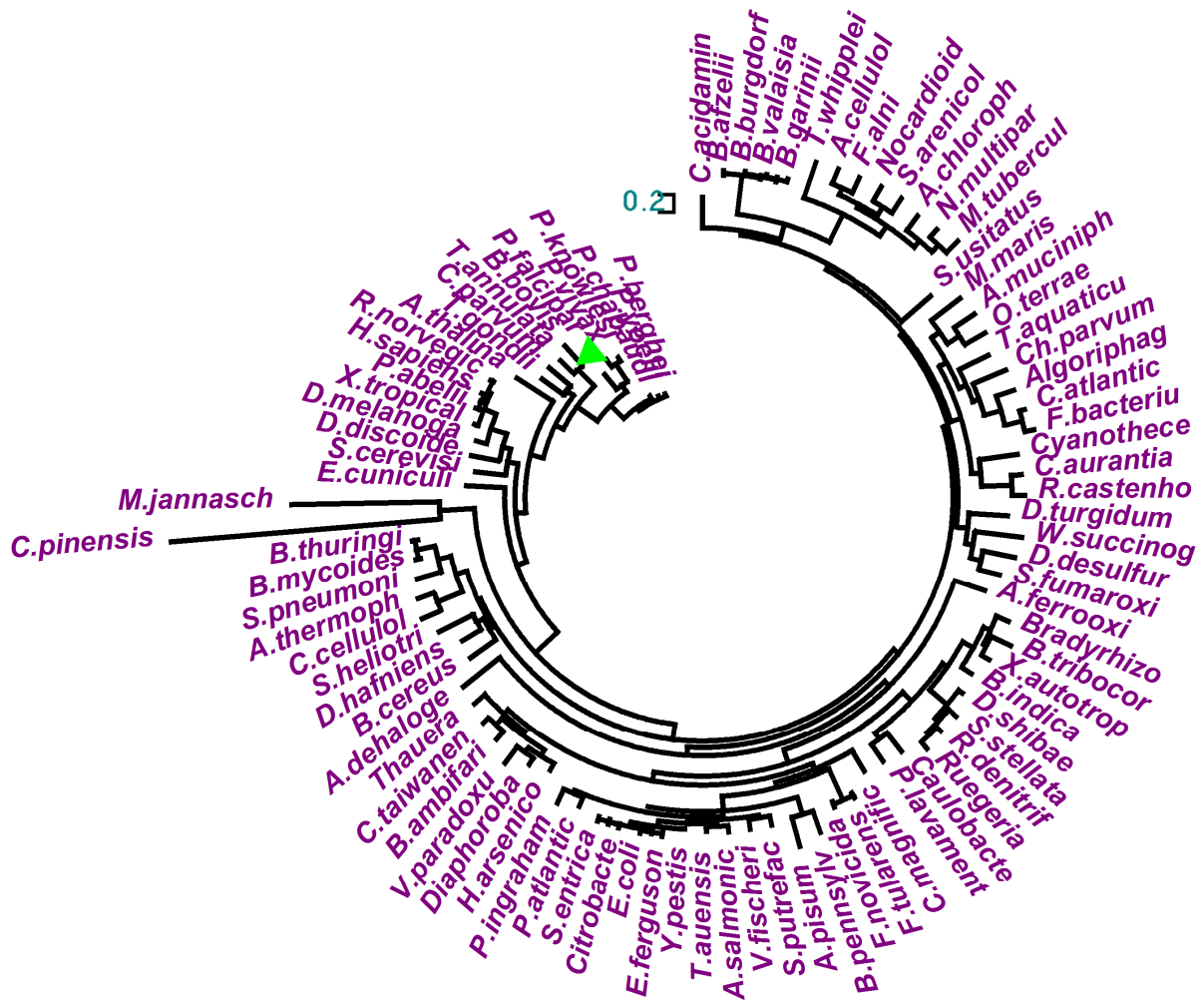

TYR

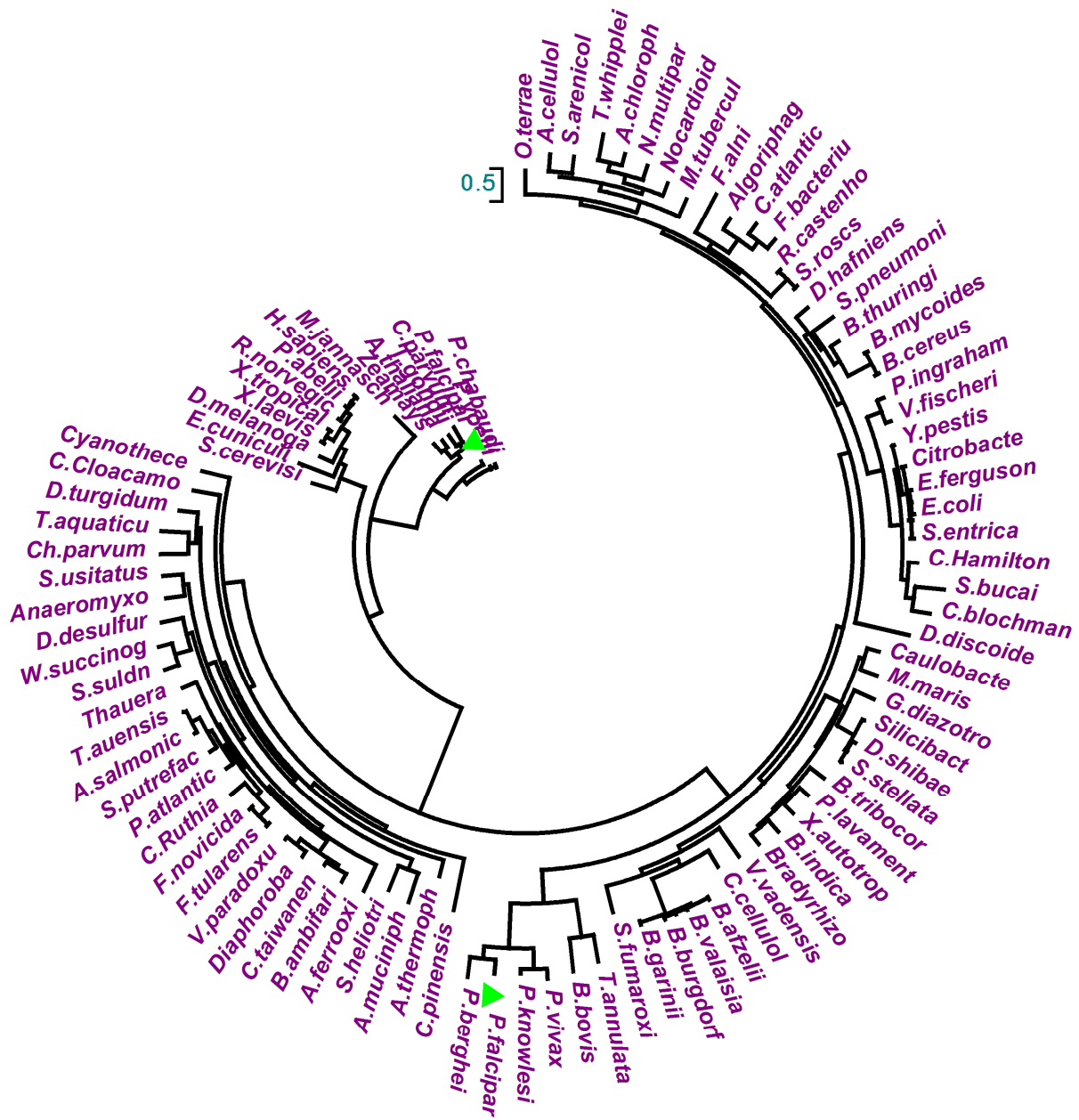

TRP

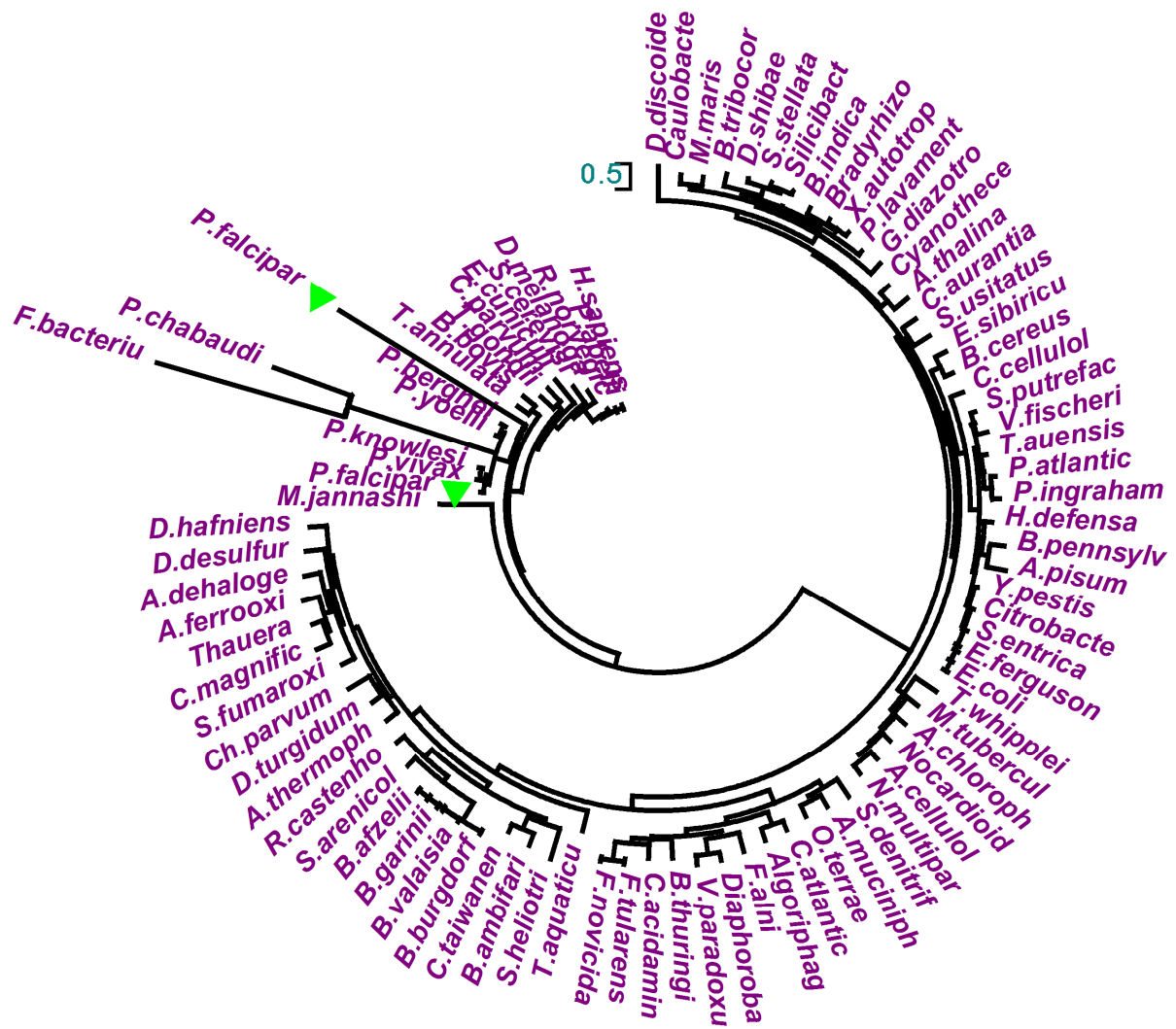

VAL

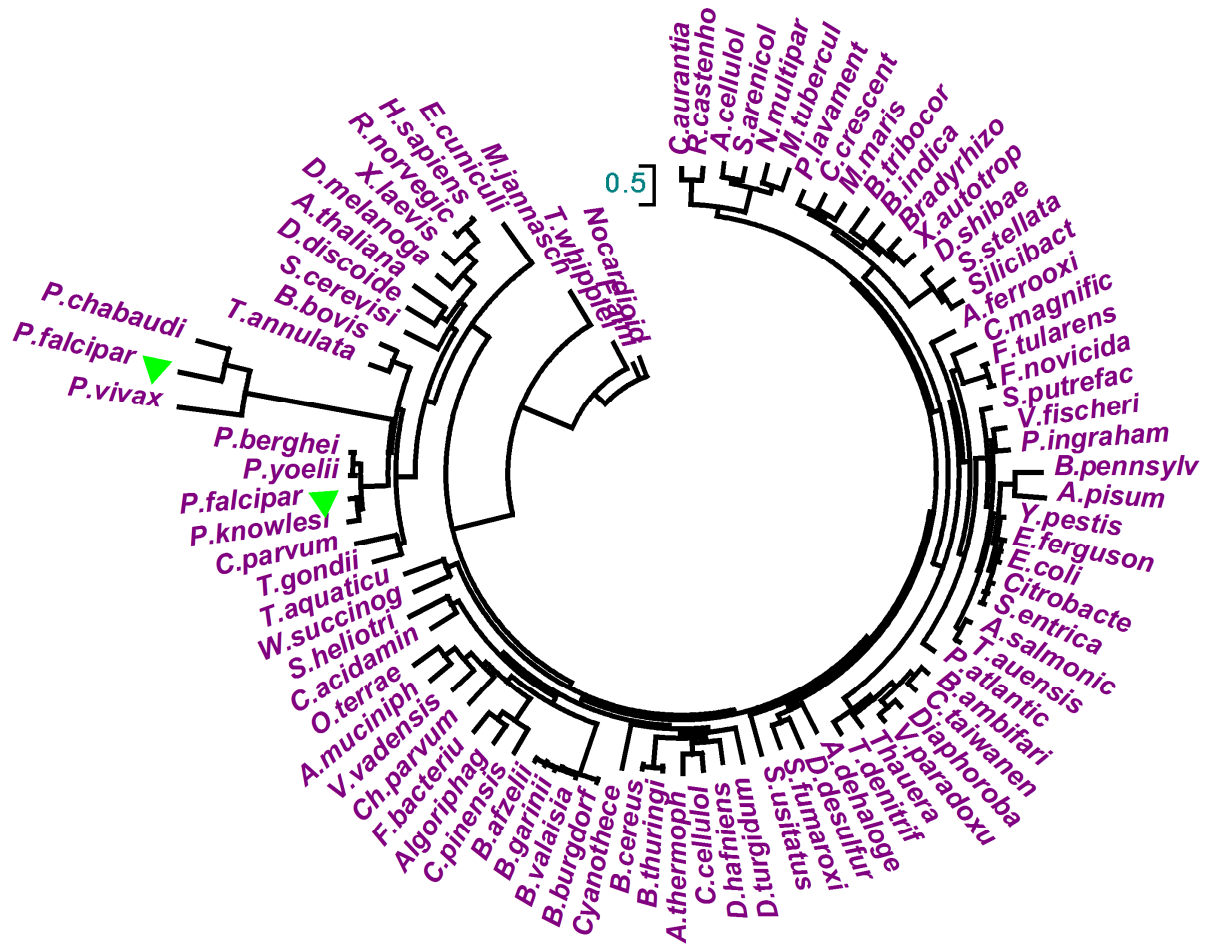

Supplement: Additional file 2 — Phylogenetic trees of aaRSs from P. falciparum. The evolutionary tree was constructed by the method PHYML using the MEGA 4.0. P. falciparum aaRSs are labeled green triangles. 102 species considered for the evolutionary analysis are taken from the three domains of life viz. P. berghei, P. chabaudi, P. falciparum, P. knowlesi, P. yoelii, P. vivax, H. sapiens, M. tuberculosis, D. discoidium, M. jannaschii, R. norvegicus, C. parvum, B. bovis, S. cerevisiae, D. melanogaster, Y. pestis, T. aquaticus, S. pneumoniae, S. entrica, E. coli, A. thaliana, A. pisum, A. salmonicida, B. cereus, B. thuringiensis, B. afzelii, B. burgdorferi, B. garinii, B. valaisiana, Bradyrhizobium, B. pennsylvanicus, C. acidaminovorans, H. defensa, C. taiwanensis, E. fergusonii, F. bacterium, F. novicida, F. tularensis, F. alni, G. tenuistipitata, H. arsenicoxydans, A. cellulolyticus, A. chlorophenolicus, A. ferrooxidans, Algoriphagus, A. muciniphila, Anaeromyxobacter, A. thermophilum, B. ambifaria, B. indica, B. mycoides, B. taurus, B. tribocorum, C. atlanticus, Caulobacter, C. aurantiacus, C. cellulolyticum, Citrobacter, C. pinensis, C. Ruthia, Cyanothece, D. desulfuricans, D. hafniense, Diaphorobacter, D. shibae, D. turgidum, E. cuniculi, E. lenta, E. ruminantium, Exiguobacterium, G. diazotrophicus, Geobacillus, M. maris, N. multipartita, Nocardioides, O. terrae, P. abelii, P. atlantica, P. denitrificans, P. ingrahamii, P. lavamentivorans, R. castenholzii, S. arenicola, S. fumaroxidans, X. autotrophicus, V. vadensis, V. paradoxus, T. whipplei, T. auensis, S. stellata, Ch. parvum, S. heliotrinireducens, Silicibacter, S. putrefaciens, S. usitatus, Thauera, X. laevis, Theileria annulata, Vibrio fischeri, W. succinogenes, X. tropicalis, Zeamays. [file 1471-2164-10-644-S2.PDF]
